# Restriction-modification mediated barriers to exogenous DNA uptake and incorporation employed by *Prevotella intermedia*

**DOI:** 10.1371/journal.pone.0185234

**Published:** 2017-09-21

**Authors:** Christopher D. Johnston, Chelsey A. Skeete, Alexey Fomenkov, Richard J. Roberts, Susan R. Rittling

**Affiliations:** 1 Department of Immunology and Infectious Disease, Forsyth Institute, Cambridge, United States of America; 2 Harvard School of Dental Medicine, Boston, MA, United States of America; 3 New England Biolabs, Ipswich, MA, United States of America; Universität Stuttgart, GERMANY

## Abstract

*Prevotella intermedia*, a major periodontal pathogen, is increasingly implicated in human respiratory tract and cystic fibrosis lung infections. Nevertheless, the specific mechanisms employed by this pathogen remain only partially characterized and poorly understood, largely due to its total lack of genetic accessibility. Here, using Single Molecule, Real-Time (SMRT) genome and methylome sequencing, bisulfite sequencing, in addition to cloning and restriction analysis, we define the specific genetic barriers to exogenous DNA present in two of the most widespread laboratory strains, *P*. *intermedia* ATCC 25611 and *P*. *intermedia* Strain 17. We identified and characterized multiple restriction-modification (R-M) systems, some of which are considerably divergent between the two strains. We propose that these R-M systems are the root cause of the *P*. *intermedia* transformation barrier. Additionally, we note the presence of conserved Clustered Regularly Interspaced Short Palindromic Repeat (CRISPR) systems in both strains, which could provide a further barrier to exogenous DNA uptake and incorporation. This work will provide a valuable resource during the development of a genetic system for *P*. *intermedia*, which will be required for fundamental investigation of this organism’s physiology, metabolism, and pathogenesis in human disease.

## 1. Introduction

*Prevotella intermedia* is a major oral pathogen associated with endodontic infections, where it is often the most frequently identified species[1], and periodontal infections, where it is a member of the orange complex [2], supporting destructive inflammatory disease. In addition, *P*. *intermedia* is increasingly implicated in extra-oral disease including nasopharyngeal and intra-abdominal infections and has been shown to colonize the respiratory tract in association with chronic bronchitis [3], severe bacteremic pneumococcal pneumonia [4] and cystic fibrosis lung infections [5]. Nevertheless, the virulence mechanisms employed by *P*. *intermedia* during human infections remain ill-defined and poorly characterized, largely due to this pathogens complete lack of genetic accessibility. Functional genetic tools, such as plasmids or transposons, to permit genetic manipulation of *P*. *intermedia* and fundamental examination of its physiology, metabolism, or pathogenesis are currently unavailable, while those developed for related bacterial species appear incompatible or non-functional.

Previously, we identified a new virulence mechanism utilized by *P*. *intermedia* capable of efficiently disabling and killing infiltrating neutrophils, offering “bystander” protection to other pathogens and allowing persistent infections to become established [6]. To delineate the genes responsible for production of this virulence factor, we made numerous attempts to transform *P*. *intermedia* and generate isogenic mutants, but none were successful. All efforts to generate *P*. *intermedia* isogenic mutants have also been unsuccessful in several other laboratories [7]. Further attempts in our lab to use an *E*. *coli*/*Prevotella* shuttle vector, constructed from a small native plasmid previously isolated from *P*. *intermedia* strain YHBi [8], also failed to transform *P*. *intermedia*. The concurrent failure of this plasmid despite the presence of compatible replicative machinery led us to consider that underlying barriers to exogenous DNA were inhibiting transformation. Bacteria have evolved multiple defense mechanisms to discriminate self from non-self DNA and to specifically degrade DNA that is perceived as foreign. These mechanisms normally function as a cellular defense from invading bacteriophage but concomitantly form an active barrier to human-made DNA during genetic engineering. At least three types of these defense systems are known: 1) Restriction-Modification (R-M), 2) Clustered Regularly Interspaced Short Palindromic Repeat (CRISPR-Cas) and 3) DNA Degradation (DND) systems.

R-M systems are the most studied to date [9] and operate, with exceptions, via two enzymatic activities, a restriction endonuclease (REase) and a modification methyltransferase (MTase). Each restriction endonuclease recognizes and cuts a specific DNA target sequence on invading DNA, whereas the methyltransferase protects the same target sequence within the host’s genome by addition of a methyl (CH_3_) group, which marks it as self [10]. R-M defenses work by recognizing the methylation status of incoming DNA and degrading inappropriately (non-self) methylated target sequences. Functioning alongside R-M systems are the CRISPR-Cas systems, which operate as a heritable immunological memory of past exposures to specific phages and plasmids. CRISPR-Cas mediated defense is a multistep process where small fragments of foreign nucleic acids are recognized as non-self and incorporated into the host genome between short DNA repeats, known as a CRISPR array. Subsequently, these fragments, now spacers within the array, are used as RNA guiding molecules for an endonuclease complex which recognizes and destroys DNA containing these sequences. DND systems, more recently identified and less widespread, represent a novel host-specific restriction system associated with DNA phosphorothioate modification, which also allows differentiation between self and non-self DNA[11]. Phosphorothioates (or S-oligos) are a variant of normal DNA in which one of the non-bridging oxygen atoms is replaced by a sulfur molecule [12]. DND sulphur-based modification constitutes an additional defense mechanism whereby a specific exogenous DNA sequence, unmodified by phosphothiolation, is targeted for destruction by a host-specific restriction system. Each of these genetic defense systems can operate in isolation or in concert [13] to form effective barriers to human-made DNA during genetic engineering. Here, to facilitate development of a genetic system for *P*. *intermedia*, we sought to define and characterize the underlying genetic defense systems, and their exact targets, present in two of the most widespread and commonly used laboratory strains, *P*. *intermedia* ATCC-25611 and *P*. *intermedia* 17.

## 2. Materials and methods

### 2.1. Bacterial strains, culture conditions and nucleic acid isolation

*Prevotella intermedia* ATCC-25611 (obtained from the American Type Culture Collection) and *Prevotella intermedia* 17 [14] (originally isolated and obtained from the laboratory of Prof. Ann Progulske-Fox) are maintained within the Forsyth Institute Culture Collection. Cultivation of *Prevotella intermedia* strains was performed anaerobically (85% N_2_, 10% H_2_, and 5% CO_2_) using either tryptic soy (TS) agar containing 5% sheep blood (blood agar plates, BAP) (Becton Dickinson, Cat # 221239) for 3–5 days or TS liquid media supplemented with 2.5% yeast extract (Gibco), L-cysteine HCl (0.4 mg/ml), hemin (5 μg/ml) and vitamin K1 (1 μg/ml). For liquid cultivation *P*. *intermedia* was first grown for 72 hours using solid BAP. A single colony was then inoculated to 10 ml pre-reduced TSB, grown until stationary phase (OD_600nm_ 1.8) and subsequently inoculated at an OD_600nm_ of 0.01 to 25 ml TSB and incubated for up to 72 hours for DNA isolation. *E*. *coli* ER2796 [15] was used as a cloning host within this study and was grown at 37°C in Luria–Bertani media supplemented with kanamycin (50 μg/ml) alone or in addition to ampicillin (150 μg/ml) after transformation. Genomic DNA from *Fusobacterium nucleatum* strain ATCC 25586 was obtained from Dr. Susan Bullman, Dana Farber Cancer Institute, USA. Reagents were purchased from Sigma unless stated otherwise. Isolation of genomic DNA from *P*. *intermedia* strains was performed using the QIAamp DNA mini kit (Qiagen) after growth to mid-exponential phase (OD_600nm_ 1.0). Plasmid DNA was isolated from *E*. *coli* ER2796 using a QIAprep spin miniprep kit (Qiagen) as per the manufacturer's instructions.

### 2.2. Single Molecule Real Time (SMRT) sequencing and methylome analysis

Genomic samples of *P*. *intermedia* ATCC-25611 and *P*. *intermedia* 17 were prepared for SMRT sequencing following standard SMRTbell template preparation protocols for base modification detection (www.pacb.com). Genomic DNA samples were sheared to an average size of 20 kbp via G-tube (Covaris; Woburn, MA, USA), end repaired and ligated to hairpin adapters. SMRT sequencing was carried out on the PacBioRSII (Pacific Biosciences; Menlo Park, CA, USA) with P6/C4 chemistry at the Johns Hopkins Deep Sequencing & Microarray Core Facility, using standard protocols for small insert SMRTbell libraries. All samples achieved ~500× average sequencing coverage across the genome. Sequencing reads were processed and mapped to the respective reference sequences using the BLASR mapper (http://www.pacbiodevnet.com/SMRT-Analysis/ Algorithms/BLASR) and the Pacific Biosciences’ SMRTAnalysis pipeline (http://www.pacbiodevnet.com/ SMRT-Analysis/Software/SMRT-Pipe) using the standard mapping protocol. Interpulse durations were measured and processed for all pulses aligned to each position in the reference sequence. The principle of base modification detection using SMRT sequencing by synthesis has been detailed previously [16]. To identify modified positions, we used Pacific Biosciences’ SMRTanalysis v2.3.0 patch 5, which uses an *in silico* kinetic reference and a t-test based kinetic score detection of modified base positions.

### 2.3. Bisulfite sequencing

Site directed bisulfite sequencing was performed during identification of 5-methylcytosine DNA methylation. Genomic sequence analysis was performed using the Seqbuilder and Seqman programs of the DNASTAR software package (DNASTAR, Madison, WI). Four ~500 bp regions of the *P*. *intermedia* ATCC-25611 genome, densely populated with putative 5-methylcytosine recognition motifs identified by REBASE or from SMRT sequencing basemod analysis, were selected *in-silico* for primer design. Site-directed bisulfite sequencing was performed using primers designed with MethPrimer [17], an open access design program for methylation mapping. Primers are listed in **S1 Table**. All bisulfite treatments of *P*. *intermedia* genomic DNA were performed using the EpiMark Bisulfite Conversion Kit (NEB). Bisulfite PCR reactions were performed using ~100 ng of bisulfite-treated DNA, and TaKaRa EpiTaq HS (Clontech Laboratories, Inc., Mountain View, CA, USA). Amplification consisted of an initial denaturation step at 98°C for 20 sec followed by 40 amplification cycles (98°C for 10 sec, 50°C for 35 sec, and 72°C for 1 min/kb + 5min final extension seconds). Amplified PCR products were purified using the QIAquick PCR purification kit (Qiagen) and product sequencing was performed by Macrogen (Cambridge, USA).

### 2.4 Cloning, restriction analysis, and confirmation of methylated motifs by SMRT sequencing.

Individual MTase genes were cloned into the constitutive expression vector pRRS as previously described [16] and transformed into *E*. *coli* ER2796, a strain deficient in methyltransferase activity (*dam*-/*dcm*-/*hsdM-*). Primers **(S1 Table)** were designed to clone MTase genes in isolation or to splice isolated MTase genes to individual specificity subunits. All PCR reactions were carried out using the Phusion DNA Polymerase Kit (NEB) in accordance with the manufacturer's instructions. Restriction enzymes and T4 DNA ligase were purchased from New England Biolabs (NEB). Each restriction digest and T4 ligation reaction was carried out as per the manufacturer's instructions using 0.2-mL PCR tubes in a thermocycler block (Eppendorf) to ensure optimal temperature conditions. Ligation reaction mixtures were purified using the QIAquick PCR purification kit. *E*. *coli* ER2796 competent cells were generated according to the Inoue method as described previously [18] and stored at -80°C until use, while transformation was performed using the heat shock method [19]. After transformation, the genomic and plasmid DNA of individual *E*. *coli* clones were analyzed by SMRT sequencing at NEB (Ipswich, MA) as described previously [16]. Where possible, methyltransferase activity was confirmed using a restriction enzyme assay. The commercially available restriction enzymes Sau3AI, MboI, DpnI and DpnII, which are sensitive to the methylation status of the ^5’-^GATC^-3’^ motif, were utilized in DNA restriction assays as previously described [20]. Genomic or plasmid DNA (0.5 μg) were incubated with 1U of restriction enzyme for 37°C for 1 hour as per the manufacturer’s instructions (NEB). The resulting restriction patterns, obtained after separation on a 1% agarose gel, were compared with unmethylated DNA controls.

### 2.5. Bioinformatic analyses

Comparative bioinformatic analyses of *P*. *intermedia* ATCC-25611F and *P*. *intermedia* 17F was performed using the Rapid Annotation using Subsystem Technology, RAST server version 2.0 [21]. Homology searches were performed using the Basic Local Alignment Search Tool (BLASTp and BLASTn) through the National Centre for Biotechnology Information (NCBI) website (available at http://www.ncbi.nlm.nih.gov/). Both functional and sequence based analysis were performed using default settings. R-M system gene annotation was performed as previously described [22], using the SEQWARE computer resource, a BLAST-based software module in combination with the curated restriction enzyme database (REBASE) [23]. Prediction was supported by sequence similarity, presence, and order of predictive functional motifs, and the known genomic context and characteristics of empirically characterized R-M system genes within REBASE. Predicted R-M system genes were named in accordance with the standard nomenclature [24]. A predicted MTase was designated as an orphan MTase if we were unable to detect an REase gene with the same target site in its neighborhood (less than 10 genes away, based on genomic coordinates) as described previously [25]. Genomes were screened for the presence of Clustered regularly interspaced short palindromic repeats (CRISPRs) using a combination of CRISPRFinder [26], CRISPRdetect [27], and CRISPROne (http://omics.informatics.indiana.edu/CRISPRone) while protospacer targets were analyzed using the CRISPRTarget [28] server. Transcriptional promoters and Rho-independent terminators were predicted using the BDGP Neural Network Promoter Prediction program (www.fruitfly.org/seq_tools/promoter.html) and the ARNold Web server (http://rna.igmors.u-psud.fr/toolbox/arnold/) respectively. Additional genome analysis was performed using the PHASTER (PHAge Search Tool – Enhanced Release) server [29], the online tool Weblogo (http://weblogo.berkeley.edu/logo.cgi) [30] to analyze the conservation of the associated repeats, and Mfold (http://unafold.rna.albany.edu/?q=mfold/RNA-Folding-Form) for secondary structure prediction of repeats [31].

## 3. Results and discussion

### 3.1. SMRT genome sequencing of *P*. *intermedia* ATCC-25611F and Strain 17F

**Fig 1:**
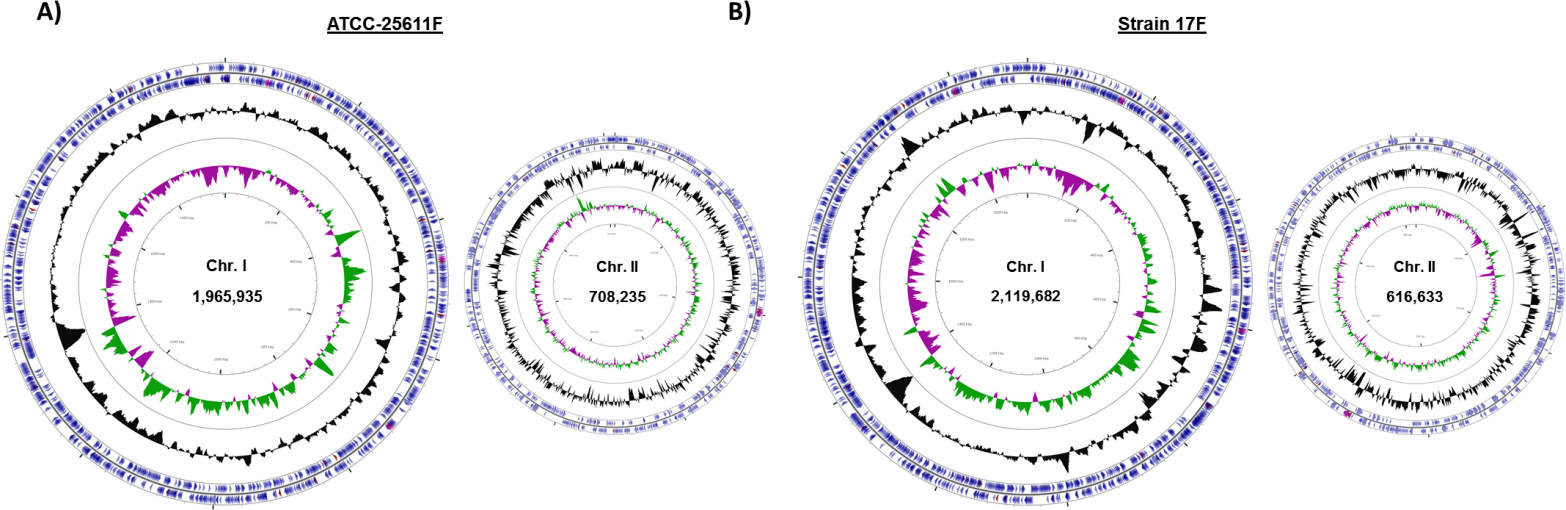
Complete genome map of *Prevotella intermedia* strains ATCC-25611F and 17F, represented by CGView. Circular maps of the two chromosomes (I and II) present within **A)** *Prevotella intermedia* ATCC-25611F (GenBank: CP019300 and CP019301) and **B)** *Prevotella intermedia* strain 17-F (GenBank: CP019302 and CP019303). The outermost two circles indicate coding DNA sequences (CDSs) on the plus and minus strands, respectively. The GC content and GC skew (Green GC+ and purple GC-) are shown in the third and fourth circles (moving towards the center), respectively.

Based on their widespread use and inclusion in numerous studies on the virulence of *Prevotella intermedia* species, we selected two common strains, *P*. *intermedia* ATCC-25611 and *P*. *intermedia* 17, for genome/epigenome sequencing and subsequent analysis. The whole genome nucleotide sequences of ATCC-25611 and strain 17 were determined using the SMRT sequencing. For ATCC-25611, *de novo* assembly of 107,365 reads with a mean length of 17,680 bp using the Hierarchical Genome Assembly Process (HGAP) algorithm in the SMRT Analysis version 2.3 resulted in two closed circular chromosomes of 1,965,935-bp (42.8% G+C content) and 708,235-bp (45.3% G+C content) in size with average coverages of 519.5× and 406.36×, respectively **(Fig 1A)**. In the case of strain 17, *de novo* assembly of 105,014 reads with 22,622-bp mean read length also produced two closed circular chromosomes 2,119,682-bp (43.1% G+C content) and 616,633-bp (44.6% G+C content) in size with average coverages of 591.5x and 451.3x coverage respectively **(Fig 1B)**. Due to previous iterations of these genomes being available we have re-designated our genomes and corresponding strains as ATCC-25611F and Strain 17F, where F indicates Forsyth Institute culture collection. The genomes were annotated by RAST (Rapid Annotation using Subsystem Technology) version 2.0 [21], and complete sequences and annotations **(S1 Fig)** have been submitted to NCBI under Bioproject ID: PRJNA313956 (*P*. *intermedia* ATCC25611F: Biosample SAMN04529094, Accessions CP019300 and CP019301 for Chromosome I and Chromosome II respectively. *P*. *intermedia* 17F: Biosample SAMN04529095, Accessions CP019302 and CP019303 for Chromosome I and Chromosome II respectively).

### 3.2. Methylome analysis and delineation of *Prevotella intermedia* R-M systems

R-M systems and their cognate recognition sequences are species-specific and often even strain specific; requiring empirical analysis for each strain of interest [10]. The restriction enzyme database, REBASE, already maintains individual genome sequence data for *P*. *intermedia* ATCC-25611 (incomplete genome, 23 contigs, Genbank: NZ_JAEZ00000000.1) and Strain 17 (complete genome, Genbank: Chromosome I:NC_017860.1/CP003502.1 and chromosome II:NC_017861.1/CP003503.1), however, no empirical information on DNA methylation motifs has been available for either strain. To rectify this and effectively characterize the R-M systems present in these *P*. *intermedia* strains, we first determined the methylome of each through analysis of the SMRT sequencing kinetics. Base modifications were analyzed **(S2 Table)** and modified sequence motifs for each strain are summarized in **Table 1**and **Table 2**.

**Table 1:** Summary of restriction-modification and orphan methyltransferase systems of *Prevotella intermedia* ATCC-25611F.

Type	System	Predicted Recognition Sequence/s ^a^	Activity status	Gene/s	Name	Location	Coordinates
I	R-M	C**A**GN_6_TTG **A**GYN_5_RTTC ^b^ DAGYN_5_CTG ^b^	Active, rearranging.	C	C.Pin25611FORF7790P	Chromosome I	1804593-1804916 c
				R	Pin25611FORF7790P	Chromosome I	1801709-1804552 c
				M	M.Pin25611FORF7790P	Chromosome I	1799511-1801064 c
				S	S1.Pin25611FORF7790P	Chromosome I	1796978-1797178
				S	S2.Pin25611FORF7790P	Chromosome I	1797150-1797518
				S	S3.Pin25611FORF7790P	Chromosome I	1797527-1798072
				S	S4.Pin25611FORF7790P	Chromosome I	1798281-1798463 c
				S	S5.Pin25611FORF7790P	Chromosome I	1798692-1799186 c
II	R-M	GG**A**TG	Active	R	Pin25611FIP	Chromosome II	255879-257306 c
				M	M1.Pin25611FI	Chromosome II	257299-258365 c
				M	M2.Pin25611FI	Chromosome II	258362-259297 c
II	R-M	G**A**TC	Active	R	Pin25611FII	Chromosome I	1005118-1005939
				M	M.Pin25611FII	Chromosome I	1004114-1005109
II	Orphan	G**C**WGC ^c^	Active	C	C.Pin25611FIIIP	Chromosome I	376745-376975
				M	M.Pin25611FIII	Chromosome I	377075-378157
II	R-M	GAATTC	Inactive	R	Pin25611FORF1345P	Chromosome I	304555-305658
				M	M.Pin25611FORF1345P	Chromosome I	303512-304483
II	Orphan	G**A**GN_4_TAC ^b^	Active	C	C.Pin25611FIVP	Chromosome II	604487-604717 c
				M	M.Pin25611FIV	Chromosome II	602445-604472 c
				S	S.Pin25611FIVP	Chromosome II	601045-602448 c
IV	R-M	Unknown	Unknown, presumed active.	R	Pin25611McrBP	Chromosome II	210423-212825
				R	Pin25611McrCP	Chromosome II	212815-214140

Systems were designated as Type I, II or IV based on gene characterization through REBASE and structural organization of operons, and assigned as orphan MTases if we were unable to detect a cognate REase with the same target site in its vicinity (less than 10 genes away, based on genomic coordinates). Predicted recognition sequences and the modifications present were determined using a combination of REBASE analysis, SMRT basemod detection, bisulfite sequencing, cloning and transformation of MTases to the methyl-deficient *E*. *coli* ER2796 and re-analysis by SMRT sequencing or restriction enzyme assays if commercially available REase enzymes were available.

*a*: The modified base within each motif is shown in bold, while the modified base on the complementary strand is underlined.

*b*: Novel recognition sequences.

*c*: Detected only after TET-assisted SMRT library preparation. Activity status was assigned based on the presence or absence of methylation within the predicted recognition motif during SMRT or bisulfite sequencing. Gene assignments, nomenclature and genome coordinates were submitted to REBASE for public release.

**Table 2:** Summary of restriction-modification and orphan methyltransferase systems of *Prevotella intermedia* 17F.

Type	System	Predicted Recognition Sequence ^a^	Activity status	Gene/s	Name	Location	Coordinates
I	R-M	G**A**GN_6_TGG	Active	R	Pin17FIVP	Chromosome I	221503-224787
				M	M.Pin17FIV	Chromosome I	224809-227148
				S	S.Pin17FIV	Chromosome I	227277-228296
I	R-M	G**A**GN_6_TTA	Active	C	C.Pin17FVP	Chromosome II	582519-582764 c
				R	Pin17FVP	Chromosome II	579636-582479 c
				M	M.Pin17FV	Chromosome II	576352-577905 c
				S	S.Pin17FV	Chromosome II	574124-575347 c
				S	S2.Pin17FORF12055P	Chromosome II	573103-573579
II	R-M	GG**A**TG	Active	R	Pin17FI	Chromosome II	408163-409563 c
				M	M1.Pin17FI	Chromosome II	409556-410623 c
				M	M2.Pin17FI	Chromosome II	410620-411555 c
II	Orphan	G**A**TC	Active	M	M1.Pin17FII	Chromosome I	1675829-1676704 c
					M2.Pin17FIIP	Chromosome I	1677572-1678549 c
II	R-M	GGNY**A**G ^b^	Active	RM	Pin17FIII	Chromosome II	22231-25584
		CCNY**A**G ^b^ GGYG**A**B ^b^			Pin17FORF9595	Chromosome II	25608-26525 c
II	Orphan	Putatively B**A**	Inactive	M	M.Pin17FORF2975P	Chromosome I	681435-682265
II	Orphan	Putatively B**A**	Inactive	M	M.Pin17FORF8660P	Chromosome I	1900067-1900846
II	R-M	Unknown	Ambiguous	R	Pin17FORF11110P	Chromosome II	370472-371248 c
IV	R-M	Unknown	Unknown, presumed active.	R	Pin17FMcrBP	Chromosome II	275125-276822
				R	Pin17FMcrCP	Chromosome II	276812-278140

Systems were designated as Type I, II or IV based on gene characterization through REBASE and structural organization of operons, and assigned as orphan MTases if we were unable to detect a cognate REase with the same target site in its vicinity (less than 10 genes away, based on genomic coordinates). Predicted recognition sequences and the modifications present were determined using a combination of REBASE analysis, SMRT basemod detection, cloning and transformation of MTases to the methyl-deficient *E*. *coli* ER2796 and re-analysis by SMRT sequencing or restriction enzyme assays if commercially available REase enzymes were available.

*a*: The modified base within each motif is shown in bold, while the modified base on the complementary strand is underlined.

*b*: Novel recognition sequences. Activity status was assigned based on the presence or absence of methylation within the predicted recognition motif during SMRT or bisulfite sequencing. Gene assignments, nomenclature and genome coordinates were submitted to REBASE for public release.

In bacteria, post-replicative modification of DNA by MTase enzymes results in three types of epigenetic markers: N6-methyladenine (m6A), N4-methylcytosine (m4C) and 5-methylcytosine (m5C) [32]. Initially, we detected eleven methylated motifs in the genome of ATCC-25611F, each corresponding to m6A modifications. However, our preliminary analysis of the ATCC-25611F genome identified the presence of a putative m5C methyltransferase gene (discussed below), but no obvious m5C motif was identified using Basemod analysis (Basemod: the PacBio DNA modification sequence analysis pipeline, http://www.pacb.com/). During SMRT sequencing, the RS-II platform records both the sequence of bases added and the kinetic information between successive additions, forming a sequencing trace. DNA templates containing a methylated base cause the polymerase to stall leading to a delay in the sequence trace. This kinetic information permits identification of the exact sites in the target DNA that have been methylated and the type of marker present (m6A, m4C or m5C) based on their characteristic trace [33]. While m6A and m4C methyl groups are directly involved in base pairing, the m5C methyl group is not and is instead positioned in the major groove of the nascent double-stranded DNA, which has limited contact with the DNA polymerase [34]. Accordingly, m5C modifications cause more subtle perturbations of the DNA which in turn results in smaller and less well-defined effects on the DNA polymerase dynamics. Pretreatment of DNA with TET (Ten-eleven translocation) proteins, which convert m5C to larger modified forms of cytosine including 5hmC (5-hydroxymethylcytosine, 5fC (5-formylcytosine) and 5caC (5-carboxylcytosine), has been shown to increase the magnitude of the kinetic signature during SMRT sequencing and to enhance the direct detection of 5mC motifs [35].

To enhance the detection of a potential 5mC motif in ATCC-25611F, we supplemented our data with a second Tet-assisted SMRT library preparation and sequence analysis, which indeed revealed an additional cytosine motif not present in our initial SMRT data. This was reported as G^**m5**^CAGCN_3_G by the PacBio software, but is more likely just G^m5^CAGC. In total, twelve motifs were found for ATCC-25611 compared with ten motifs in strain 17, **(S2 Table)** and all but the single m5C were predicted to be m6A methylated sequences. Interestingly, only two motifs were shared between the strains, revealing considerable heterogeneity in methyltransferase activities.

Next, we sought to delineate the MTases responsible for each motif, their genetic loci, and whether they formed part of an active R-M system. While MTases are typically described in the context of R-M activities, they can also exist as orphan MTases which lack a cognate REase partner. Such orphan MTases are often involved in cell regulation, replication, DNA repair, and population evolution[36], but should not form an active barrier to transformation during genetic engineering of *P*. *intermedia*, with the exception of the recently described Bacteriophage Exclusion (BREX) systems [37] where phage entry is restricted, but the mechanism is unknown. None of the methylases in either *P*. *intermedia* strain appear to be part of a BREX-like system. Individual R-M systems can differ with regard to target sequences, active site architecture, and reaction mechanisms [38] but all systems recognize the methylation status of target sequences on incoming DNA and degrade inappropriately methylated (non-self) DNA. There are four distinct types; Type I-III systems each function by recognizing and cutting a target sequence if it lacks an appropriate methyl group. In contrast, Type IV systems do not use a methyltransferase enzyme and instead a methyl-dependent REase cuts a target sequence if it contains a methyl group. REBASE analysis predicted the presence of multiple Type I, Type II and Type IV, but no Type III systems in *P*. *intermedia* ATCC-25611F and 17F strains. These are discussed in the context of their individual system types.

#### 3.2.1 Type I systems

Type I R–M systems consist of separate restriction, methylation and DNA sequence-recognition subunits and are typically encoded by three genes: *hsdR* encodes the restriction (R) subunit, *hsdM* encodes the modification (M) subunit and *hsdS* the recognition or specificity (S) subunit. Type I enzyme complexes are composed of 2R+2M+S subunits that can catalyze both restriction and modification activities [39] or 2M+S subunits that can modify DNA. In both cases the S subunit is responsible for DNA sequence recognition[40]. Characteristically, these systems target bipartite DNA motifs comprising two half-sequences separated by a gap[40] For example, the EcoKI Type I system recognizes AACN_6_GTGC where N = any base and is one of the best characterized to date [39].

**Fig 2:**
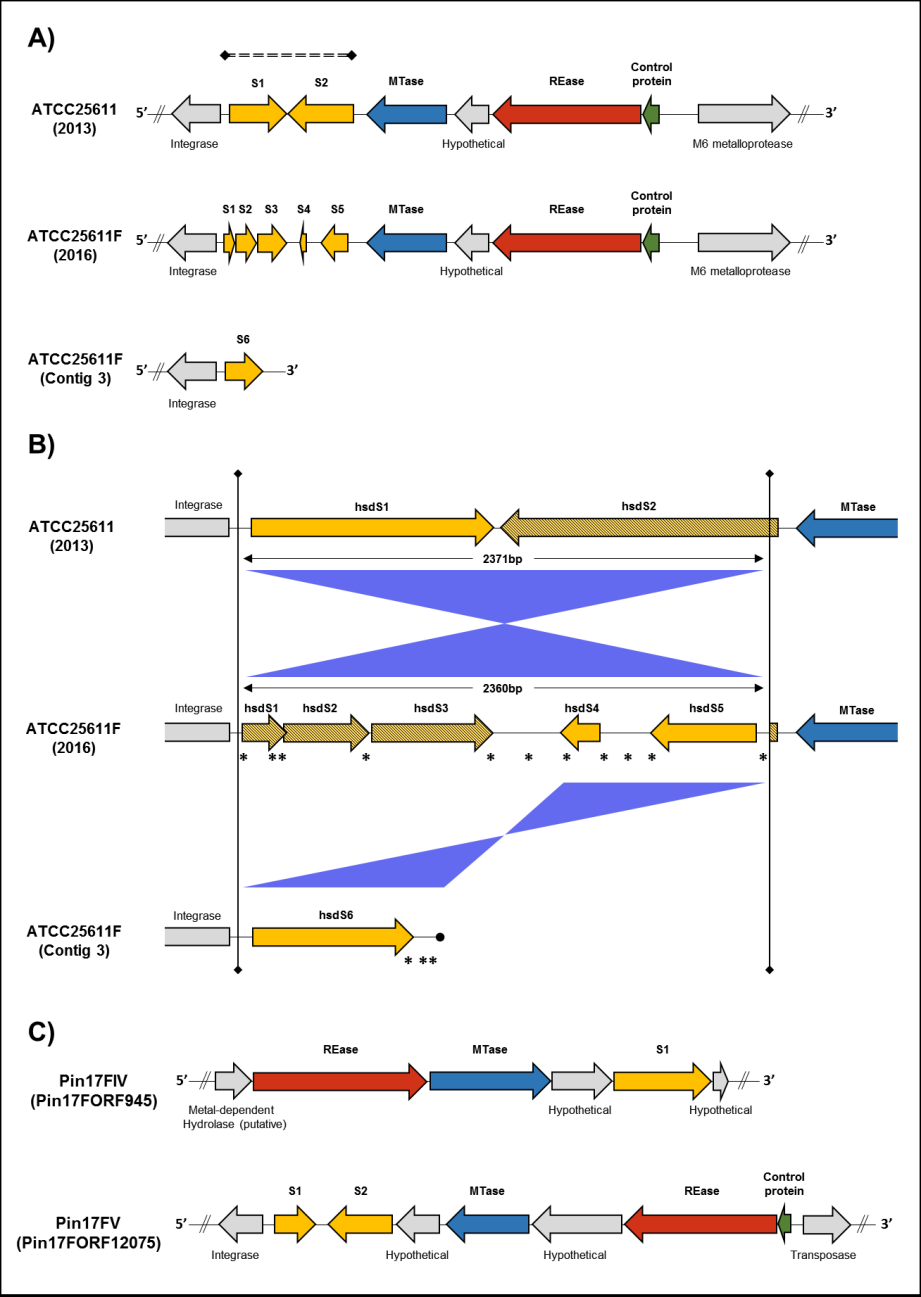
Type I restriction-modification systems of *P*. *intermedia*. **A)** A schematic diagram of gene cluster organization of *P*. *intermedia* ATCC-25611 Type I R-M system with rearranging hsdS subunits. The original incomplete draft genome of *P*. *intermedia* ATCC-25611 (2013, Genbank: NZ_JAEZ00000000.1) contains a single Type I system (upper row from right to left) with a putative control protein (green), a hsdR (red), hsdM (blue) and two hsdS (yellow) subunits (hsdS1 and hsdS2, protein accession WP_051409535 and WP_028910939, respectively). In our updated and complete *P*. *intermedia* ATCC-25611F (2016) genome sequence (middle row), a single inversion and a series of frame shift mutations within this region have transformed the original hsdS1 and hsdS2 genes into the five separate hsdS genes, hsdS1-5 (BWX39_07765, BWX39_07770, BWX39_07775, BWX39_07780 and BWX39_07785 respectively). In addition, a further truncated variant of original hsdS1 gene was also identified within a misaligned read with 50x coverage during PacBio genome sequencing (Contig 3, bottom row). The location of this dynamic region is indicated by the dashed line. **B)** Comparison of the dynamic 2.4 kb region containing the Type I R-M system hsdS genes in *P*. *intermedia* ATCC-25611 and ATCC-25611F. Here, the original reference genome of ATCC-25611 (JAEZ01000007, B129DRAFT_scaffold_4.5_C) is shown on the top row, our completed PacBio ATCC-25611F genome is shown on the middle row (Accession: CP019300) and the terminal end of the supplementary misaligned PacBio sequence read (Contig 3, 50x coverage) is shown on the bottom row. Blue bars between rows indicate regions of similarity which have inverted and are now oriented in the opposite direction, while asterisks indicate the location of frameshift deletions (compared to the JAEZ01000007 reference genome) leading to fragmentation of the original hsdS1 and hsdS2 genes. Yellow arrows indicate the hsdS1 gene and related fragments while dashed yellow arrows indicate the hsdS2 gene and related fragments. Upstream and downstream loci remain constant between genome sequences. **C)** A schematic diagram of the two Type I systems in *P*. *intermedia* 17-F. Pin17FORF945P and Pin17FORF12075P are present on chromosome I and chromosome II, respectively. Arrows represent the direction of translation and the relative sizes of open reading frames (ORFs). Putative control proteins are highlighted in green, hsdR and hsdM in red and blue, respectively, and specificity hsdS subunits are shown in yellow. Proteins not identified as part of the R-M system or those with currently unknown function are shown in grey. Exact genome coordinates of each R-M loci are indicated in **Table 1**and **Table 2**.

We identified a single Type I system in the genome of ATCC-25611F, containing a separate *hsdM* gene, a *hsdR* gene and five specificity subunit genes (*hsdS* 1-5) occupying a 2.5 kbp region directly upstream of the *hsdM* gene **(Fig 2A)**. However, the five S subunit genes are mainly fragments that are the result of *in vivo* rearrangements and probably represent one of many arrangements present in the population.

In ATCC-25611F, a single inversion of a 2371-bp fragment within the *hsdS* region, in addition to eleven individual nucleotide deletions, have transformed the two *hsdS* genes identified in the previous ATCC-25611 genome into a 2360-bp fragment with the five separate *hsdS* genes identified in our study **(Fig 2B)**. This suggests that the *hsdS* loci is a dynamic region of the *P*. *intermedia* genome, which has rearranged since the previous sequence was reported (NZ_JAEZ00000000.1). Furthermore, during assembly of our PacBio ATCC-25611F genome we identified a single 19.3kbp read, with 50x coverage, that did not align correctly with either chromosome forming a third contig unassigned to either chromosome. The majority (18.6 kbp) of this contig aligns with 99% homology to the region on chromosome-I immediately upstream of the Type I system loci, except for the terminal 0.7 kbp which overlapped with this dynamic *hsdS* loci and misaligned due to variations in the sequence **(Fig 2A and 2B)**. The *hsdS* subunit present on this unaligned contig (*hsdS6*) is a truncated version of that found in the original genome (a frameshift deletion at position 695 results in a 702 bp gene here versus the original 1,077 bp gene in ATCC-25611), which represents a further variation of *hsdS* genes which has arisen during cultivation of *P*. *intermedia*. The presence of multiple variants of this region within a single library preparation during PacBio sequencing suggests that the 2.5 kbp region is actively rearranging, even within a presumed homogenous culture preparation of *P*. *intermedia* from a single colony.

Since this is the only Type I system present on the genome, it must be responsible for the multiple Type I motifs (AGYN_5_RTTC, DAGYN_5_CTG, and CAAN_6_CTG) identified **(S2 Table)**. This variability in target motifs most likely reflects the activity of the multiple HsdS subunits and the possible rearrangements that take place within the population. A surprising feature is that the degree of modification at each site is quite high, suggesting that perhaps even the small S subunit fragments are able to combine and generate the variability in sequence recognition. However, further confirmation of this possibility would require extensive experimentation, which is beyond the scope of the current study.

Rapid intragenomic changes affecting R-M systems have been described in other species, also occurring through phase variation produced by DNA inversion [41]. Croucher *et al* identified a similar rearranging Type I system in *S*. *pneumonia*, and detected inversions within an *hsdS* locus which rearranged rapidly enough that even a single colony contained a mixture of possible sequence arrangements, each with a different target specificity [41, 42]. Genomic shuffling of *hsdS* genes therefore offers an explanation for the multiple motifs identified during methylome analysis of ATCC-25611F. In addition it also suggests a mechanism for the gap length changes within the individual motifs (N = 5 or 6), which are influenced by alterations in the length of a central conserved domain between two target recognition domains (TRDs) of HsdS subunits [40].

Interestingly, REBASE comparison indicated that the HsdS1 and HsdS2 proteins of the ATCC-25611F system matches with strong homology (59% coverage 74% identity) to the N-terminal portion of a single Type I S subunit present in *Bacteroides finegoldii* CL09T03C10 and *Bacteroides pyogenes* JCM 6294 [23], both recognizing the motif CAGN_5_CTA. In ATCC-25611F, two deletions at bp positions 150 and 182 result in a frame shift and truncation of this specificity subunit, dividing what is a single HsdS unit in these *Bacteroides* species, into the two separate *hsdS1* and *hsdS2* genes in *P*. *intermedia*, but only a single TRD is predicted to be present within the Hsd2 translated protein. This suggests that the CAG half-sequence target identified within this system (CAGN_6_TTG) likely corresponds to the TRD present within HsdS2. Additionally, the HsdS5 protein in ATCC-25611F shares homology (49% coverage, 87% identity) to the N-terminal TRD of *Listeria monocytogenes* J1-220 HsdS protein S.LmoJ1220ORF18095P, which recognizes GACN_5_RTTC. This suggests that the RTTC half-sequence target found here (AGYN_5_RTTC) may be a result of the HsdS5 protein. However, it seems likely that in other systems small S subunits can interact to form a more complicated specificity complex and more work would be needed to rigorously interpret the possible role of these small subunit fragments. Since none of the other *hsdS* genes matched with a known motif in REBASE, no further prediction is possible at this time.

In *P*. *intermedia* 17, we identified two Type I systems, each present on separate chromosomes. The first, Pin17FORF12075P, is composed of an *hsdM* and an *hsdR* gene along with two apparent *hsdS* genes, but of different length. The first is 159 aa and too short to be a genuine Type I S subunit, while the second is 517 aa and contains two TRDs characteristic of an active Type I S subunit **(Fig 2)**. The second system, Pin17FORF945P also contained an *hsdM* and *hsdR* gene but only a single *hsdS* gene, which also contained two TRDs. None of the HsdS matched to known sequences in REBASE, which prevented assignment of the GAGN_5_TGG or GAGN_6_TTA motifs identified during methylome analysis **(Table 2)** to a specific Type I system.

To follow up we selected Pin17FORF945P and cloned the *hsdM* and *hsdS* gene, but not the *hsdR*, into the constitutive expression vector pRRS for transformation to a non-methylating *E*. *coli* strain (ER2796). Total genomic and plasmid DNA from *E*. *coli* cells expressing the *hsdM* and *hsdS* subunit were tested for methylation using SMRT sequencing and surprisingly revealed several different m6A methylated motifs (GAGN_6_TRG, GAGN_6_AGG and GAGN_5_VTGAB) **(S3 Table).**

The first motif observed in recombinant *E*. *coli* (GAGN_6_TRG), matches well with the GAGN_6_TGG motif found in *P*. *intermedia* 17F, albeit with relaxed specificity, forming the degenerate R base (adenine or guanine) in the second sub-motif. This relaxed base change is likely a result of overexpression within *E*. *coli*. However, while the second and third motifs (GAGN_6_AGG and GAGN_5_VTGAB) are also clearly related on one side to the GAG motif, the other sides differ and are not present in the original *P*. *intermedia* 17F strain. The cause of these changes are not immediately clear, although it is possible that the single gene occurring between the *hsdM* and *hsdS* gene, with no predicted function from BLAST analysis, is playing an unforeseen role in specificity. Nevertheless, our results suggest that the Pin17FORF945P system is responsible for the GAGN_6_TGG motif found in the *P*. *intermedia*-17F genome.

It remained unclear whether the second motif identified in 17F (GAGN_6_TTA) was a combination of the GAG sub-motif with one of the other HsdS subunits or simply catalyzed by one or both of the Pin17FORF12075P S subunits. To clarify this, we subsequently cloned and transformed the Pin17FORF12075P system to *E*. *coli* ER2796 in two separate constructs. The first construct contained the *hdsM* of this system along with the adjacent 517 aa *hsdS* subunit gene in isolation, while the second construct contained the *hdsM* with both the proximal and the distal *hsdS* subunit genes together in their native orientation in 17F (**S1 Table)**. SMRT sequencing of these recombinant *E*. *coli* clones revealed that the proximal 517 aa *hsdS* gene alone was responsible for the GAGN_6_TTA motif identified in 17F, since no additional motif was detected by SMRT sequencing when the second *hsdS* gene was also present (**S3 Table).** Accordingly, we have determined that the Pin17FORF945P system, now called Pin17FIV is responsible for GAGN_5_TGG, while the Pin17FORF12075P system, now called Pin17FV, is responsible for GAGN_6_TTA. Both Type I systems have been assigned as active in *P*. *intermedia* 17F **(Table 2).**

In the context of genetic engineering of *P*. *intermedia* ATCC-25611F and 17F, the presence of complex and most importantly, rearranging, Type I systems are likely a considerable genetic barrier to exogenous DNA containing these unmethylated sequences.

#### 3.2.2 Type II systems

**Fig 3:**
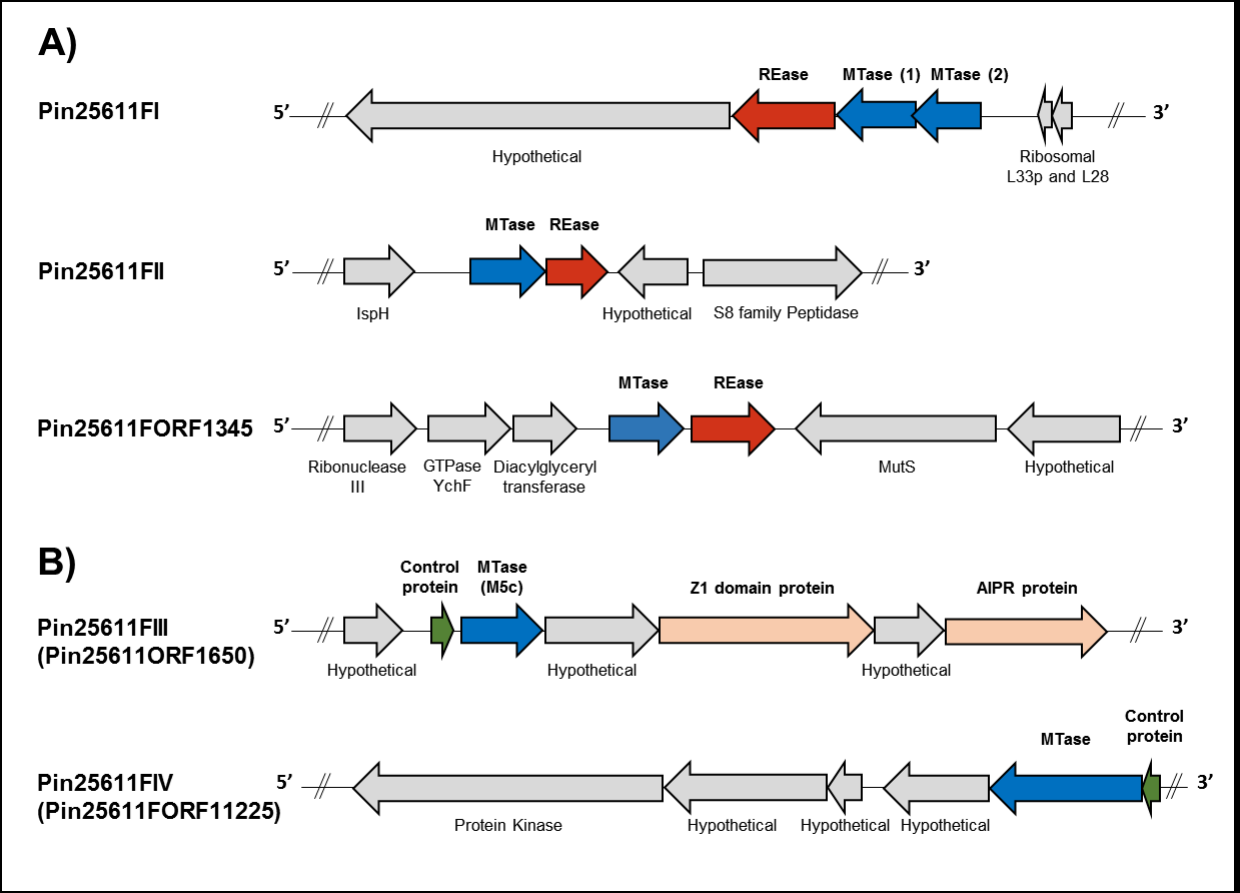
**The organization of gene clusters encoding Type II A) restriction-modification and B) orphan methyltransferase systems in *P*. *intermedia* ATCC25611F**. Arrows represent the direction of translation and the relative sizes of open reading frames (ORFs). Putative control proteins are highlighted in green, restriction endonucleases (REase) in red and methyltransferases (MTase) in blue. In addition, the Pin25611FIII system operon encodes a putative abortive infection phage resistance (AIPR) protein and a Z1-domain containing endonuclease (orange arrows), predicted by BLAST analysis. Proteins not identified as part of the R-M system or those with currently unknown function are shown in grey. Exact genome coordinates of each R-M loci are indicated in **Table 1**.

**Fig 4:**
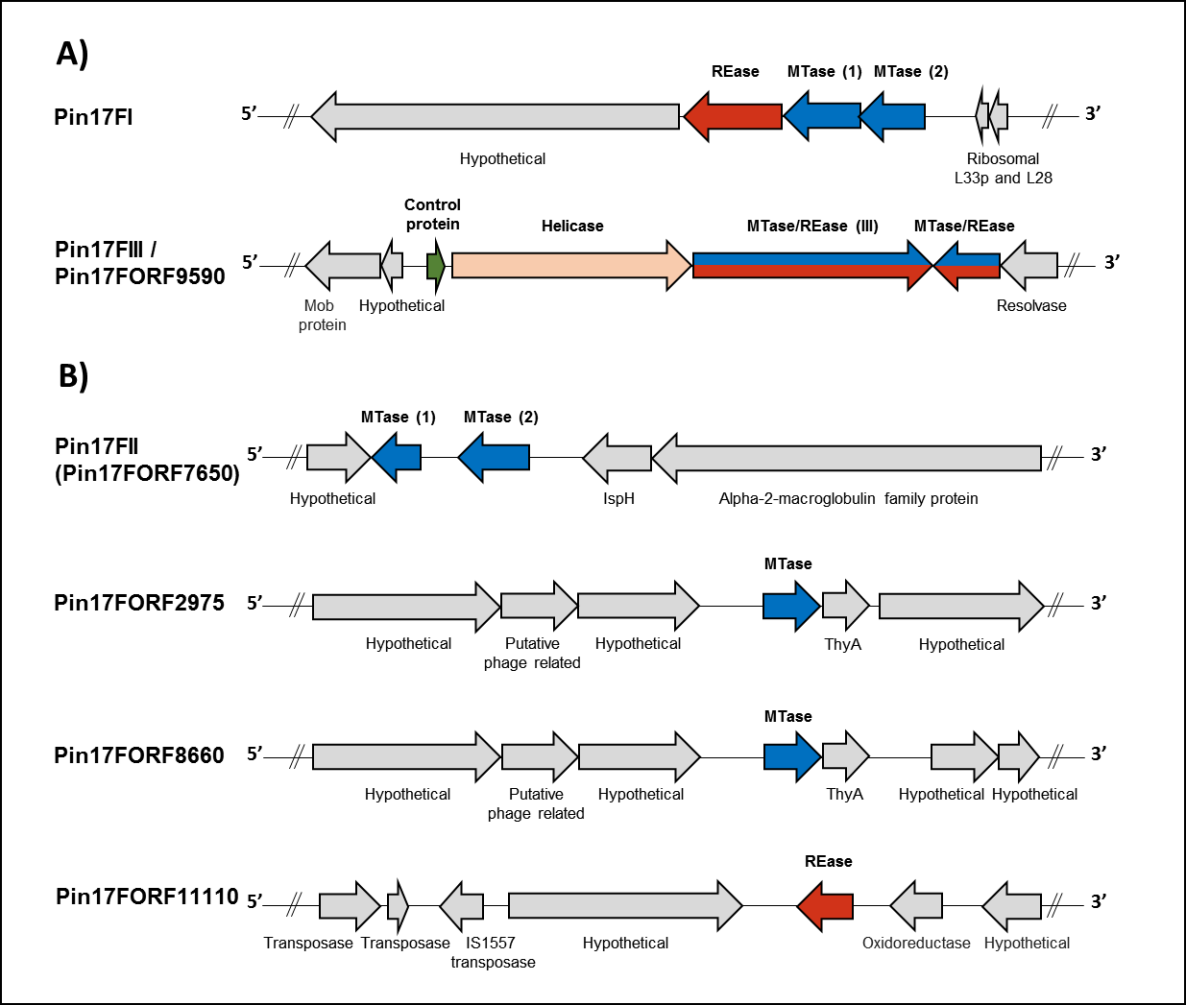
**The organization of gene clusters encoding Type II A) restriction-modification and B) orphan methyltransferase systems in *P*. *intermedia* 17F.** Arrows represent the direction of translation and the relative sizes of open reading frames (ORFs). Putative control proteins are highlighted in green, separate restriction endonucleases (REase) in red and methyltransferases (MTase) in blue. In addition, the Pin17III and Pin1FORF9590 systems are predicted to be Type IIG R-M systems, which have both REase and MTase activity present in a single polypeptide chain and are highlighted in red/blue arrows. Proteins not identified as part of an R-M system or those with currently unknown function are shown in grey with their predicted function from BLAST analysis indicated. Exact genome coordinates of each R-M loci are indicated in **Table 2**.

Type II R-M systems are for the most part composed of independent REase and MTase proteins recognizing the same sequence [43]. We identified five Type II systems present in the genomes of the ATCC-25611F and six in 17F, (**Fig 3**and **Fig 4**, respectively). We further determined the specificities of each system using a combination of REBASE prediction, cloning and SMRT sequencing of isolated MTases or restriction assays, in addition to bisulfite sequencing.

In ATCC-25611F, we were able to assign putative recognition sequences for three of the five systems based on REBASE predictions and the presence of the predicted motifs in the methylome. The first system, Pin25611FI, contains two MTase genes **(Fig 3A)** and an REase coding gene, all of which are homologs (average 65% identity) of the FokI-like system (6mA) in *Treponema succinifaciens* DSM 2489, recognizing the non-palindromic sequence GGATG/CATCC, both motifs were identified in our methylome data **(S2 Table)**.

**Fig 5:**
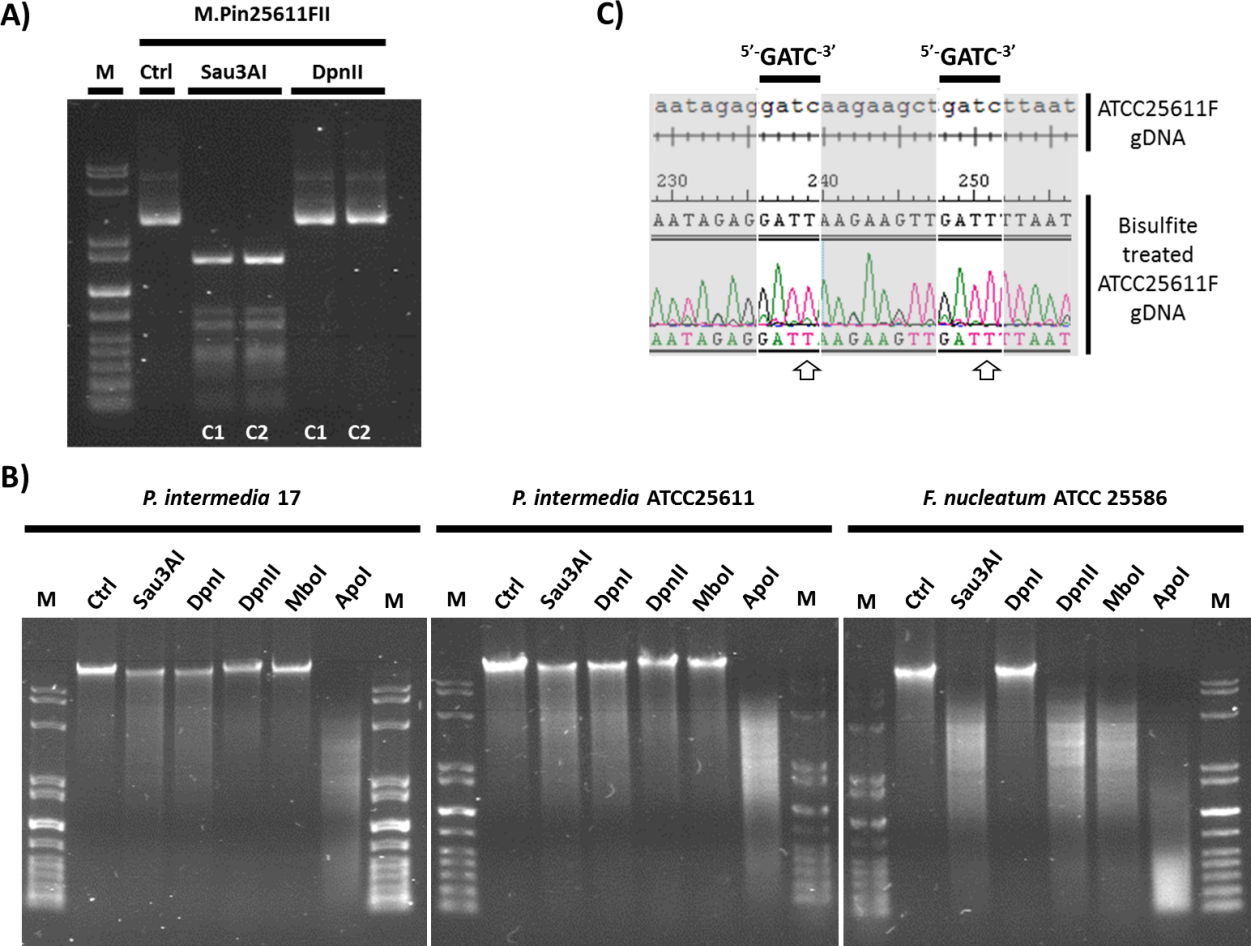
Methyltransferase activities targeting the palindromic GATC motif in *P*. *intermedia* strains. **A)** The methyltransferase associated with the Pin25611FII R-M system (M.Pin25611FII) is responsible for the G^m^ATC motif modification in *Prevotella intermedia*. The methyltransferase gene was cloned to plasmid pRRS and expressed in *E*. *coli* ER2796, deficient in methyltransferase activity. Plasmid DNA (1 μg), isolated from two separate clones (C1 and C2) of the recombinant construct, was restricted with 1U of either Sau3AI (inhibited by GAT^m^C, unaffected by G^m^ATC) or DpnII (inhibited by G^m^ATC, unaffected by GAT^m^C). Lane M, marker DNA (10kb ladder); lane 1, undigested control plasmid DNA; lane 2 and 3, Sau3AI digested plasmid DNA; lane 4 and 5, DpnII digested plasmid DNA from separate clones (C1 and C2). **B)** Restriction enzyme digestion of genomic DNA following isolation from *P*. *intermedia* 17F, *P*. *intermedia* ATCC25611F and the control *Fusobacterium nucleatum* ATCC25586. Genomic DNA (1 μg) was restricted with 1U of restriction enzymes each recognizing GATC, but differing in their methylation sensitivity, for 1 hour and resolved on a 1% agarose gel. In each gel image: Lane M, marker DNA (10kb ladder); lane 1, undigested control DNA; lane 2, Sau3AI (inhibited by GAT^m^C); lane 3, DpnI (methyl-directed endonuclease, requires G^m^ATC but inhibited by GAT^m^C); lane 4, DpnII (inhibited by G^m^ATC, unaffected by GAT^m^C); lane 5, MboI (inhibited by G^m^ATC and GAT^m^C) and lane 6, ApoI treatment (control enzyme, recognizes sequence RAATTY, where R = A or G, and = C or T). *P*. *intermedia* DNA appears to be methylated at the adenine and cytosine residues of the GATC motif, while *F*. *nucleatum* DNA is unmethylated at both residues. **C)** The absence of GAT^m5^C modification in *P*. *intermedia* DNA. Comparison and alignment of untreated ATCC25611F genomic DNA region with the same region after bisulfite conversion indicates the absence of m5C modification within ^5’-^GATC^-3’^motifs. During bisulfite treatment, unmethylated cytosine or N4-methylcytosine (m4C) is converted to uracil which is read as thymine during sequencing, while 5-methylcytosine (m5C) is not subject to deamination and remains as cytosine. White arrows indicate cytosine residues of the GATC motif converted to thymine. DNA was amplified and sequenced using primer set GATCregion1_BS and GATCregion2_BS, detailed in **S1 Table** (full sequence comparison of this and a second GATC region are shown in **S2 Fig**).

The second Type II system in ATCC-25611F, M.Pin25611FII, putatively recognized GATC based on its sequence similarity to M.Mae7806VIII and M.MboI, both of which are characterized as recognizing GATC. M.Pin25611FII is also adjacent to an REase gene with 51% identity to the known GATC-recognizing enzyme MboI. This m6A motif appears in the methylome and to confirm the Pin25611FII system as responsible, we separately cloned the MTase, transformed the methylation-deficient *E*. *coli* strain ER2796. Isolated plasmid DNA from this strain was digested with Sau3AI and DpnII to differentiate between adenine and cytosine methylation of this motif. Plasmid DNA from the cells expressing M. Pin25611FII were resistant to DpnII cleavage but not Sau3AI **(Fig 5A)**, confirming that defense against this R-M system is mediated by m6A modification of the GATC motif.

The third system, M.Pin25611FORF1345P, is predicted to encode an adenine-specific MTase homolog of EcoRI (51% identity across 99% coverage), an *E*. *coli* MTase recognizing the sequence GAATTC. Based on the location of an REase encoding gene, immediately downstream of this MTase gene, it is expected that this enzyme also targets the GAATTC motif. However, no such methylated sequence was detected during methylome analysis, suggesting that this Type II system is inactive in *P*. *intermedia*. Interestingly, the 2.6 kb loci where this R-M system is present in ATCC-25611F is absent in the genome of *P*. *intermedia* 17F.

**Fig 6:**
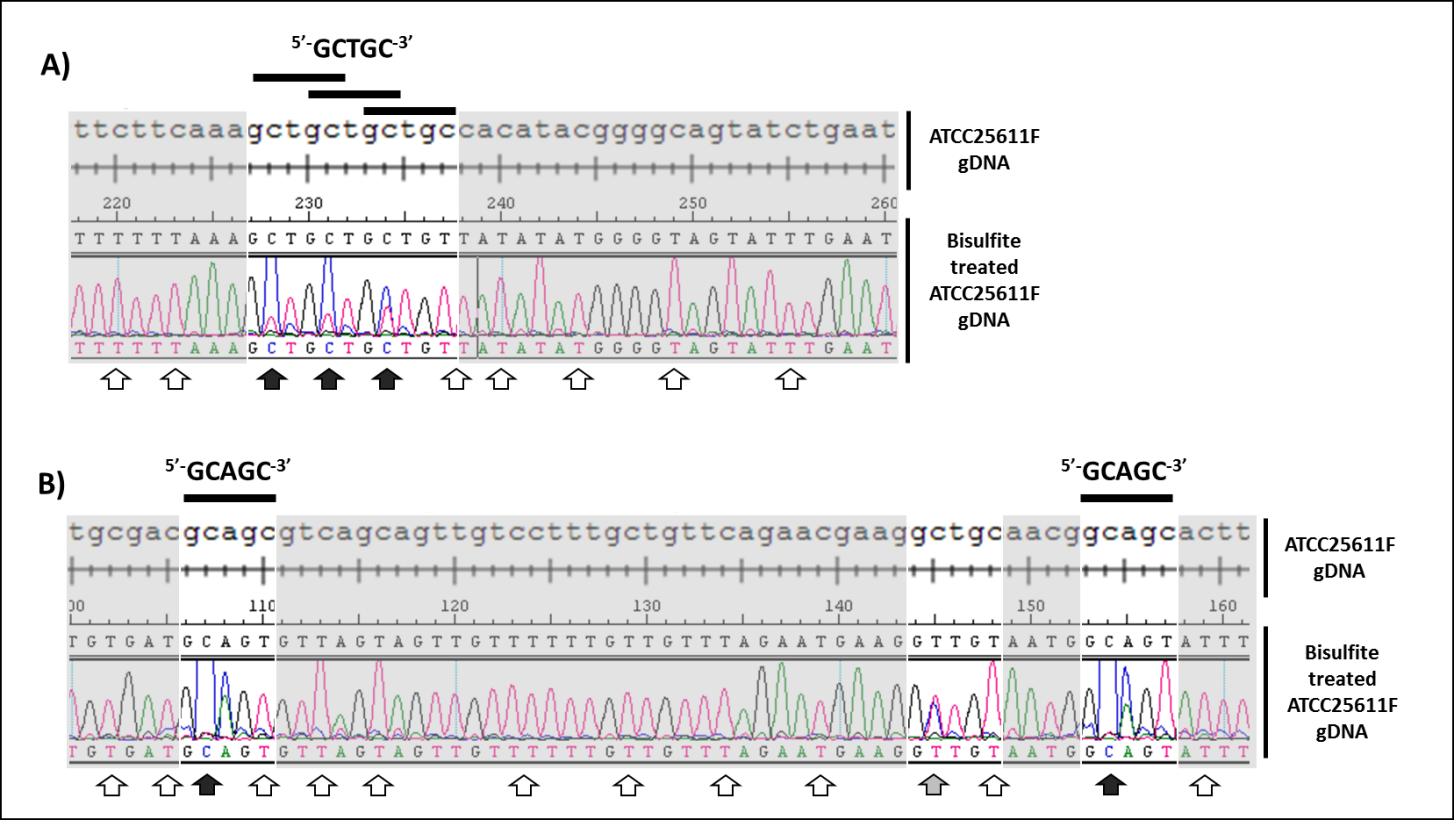
Site directed bisulfite sequencing of *P*. *intermedia* ATCC-25611F DNA identifies 5-methylcytosine (m5C) modifications of ^5’-^GCWGC^-3’^ motifs (where W = A or T). Sequence comparison and alignment of ATCC25611F genomic regions before and after bisulfite conversion. **A)** Example of region containing GCTGC motifs and **B)** example of region containing GCAGC motifs. Unmethylated cytosine residues converted to thymine during bisulfite treatment are indicated by white arrows, m5C methylated cytosines protected from deamination are indicated by black arrows (present within GCWGC motifs) while partially converted cytosine residues (shown by dual red and blue peaks in chromatogram) are indicated by grey arrow. DNA was amplified and sequenced using primer set GCWGCregion1_BS and GCWGCregion2_BS, detailed in **S1 Table** and full sequence comparison of these regions are shown in **S3 Fig**.

The remaining Type II systems in ATCC-25611F, M.Pin25611FORF11225P and M.Pin25611FORF1650P, both lacked an assignable motif based on similarity to known systems. Since M.Pin25611ORF1650P is an m5C methylase and the only one in the genome, the G^m5^CAGC motif identified during TET-assisted SMRT sequencing **(S2 Table)** was the best candidate, and we used bisulfite sequencing to confirm its recognition sequence. Bisulfite conversion leads to deamination of unmethylated cytosine residues to uracil (U), which are read as thymine (T) after PCR amplification, but leaves m5C intact allowing for identification of m5C methylation at single nucleotide resolution during sequence analysis. gDNA from two genomic regions was bisulfite-converted, amplified by PCR and sequenced. Sequencing chromatograms revealed complete bisulfite conversion of all C residues except for those at position 2 of both GCAGC and GCTGC sites **(Fig 6A and 6B)** indicating that the m5C MTase of ATCC-25611F is specific for the GCWGC motif. Nonetheless, only 66% of GCWGC sites present within amplicons (n = 11) were protected from deamination suggesting that some sites in the genome are unmethylated **(S2 Fig)**. This could be either due to partial protection from methylation by DNA-binding proteins or a result of incomplete conversion of 5mC residues to thymine during bisulfite treatment [44]. The latter is certainly possible owing to the presence of dual cytosine and thymine peaks within bisulfite converted chromatograms **(Fig 6)**. This m5C methylation may also account for the presence of the GCAGC motif called by PacBio and for which there is no candidate gene. Since m5C often gives rise to aberrant signals during PacBio sequencing we suggest that this motif is really G^m5^CWGC with the modification as indicated. We would note that this MTase gene occurs in a five-gene segment with a potential endonuclease nearby, **(Fig 3A)** and we have accordingly assigned this methylase, now called M.Pin25611III, as active in ATCC-25611F **(Table 1)**.

The Pin25611FORF11225P system contains three genes encoding a putative transcriptional regulator, an MTase, and a hypothetical protein coding gene of unknown function which directly overlaps (4 bp) with the upstream MTase on the same DNA strand **(Fig 3B)**. M.Pin25611FORF11225P has several remotely similar proteins in other organisms which recognize bipartite sequences separated by four N residues. Since such sequences with four N residues are uncommon in Type I systems it was possible that this gene actually coded for the methylase recognizing GAGN_4_TAC. To further investigate this we transformed *E*. *coli* ER2796 with three separate constructs containing; 1) the MTase gene in isolation, 2) the putative transcriptional regulator along with the MTase gene, and 3) the entire system including the downstream hypothetical gene of unknown function. Interestingly, SMRT sequencing of each revealed that only the final construct containing the entire system resulted in a GAGN_4_TAC methylated motif in *E*.*coli*, while the other two constructs did not produce any methylated motif despite confirmation of the full correct sequence in both (**S3 Table)**. This indicates that the downstream hypothetical protein contains a specificity subunit required for functionality in *P*. *intermedia* and we have reassigned this orphan system as Pin25611IV.

The R-M systems in *P*. *intermedia* 17F were considerably different from those of the ATCC strain: only two of the Type II systems found in ATCC-25611F were also found in the clinical isolate. The first system, Pin17FI, has 99% coverage and 93% identity to the genetic locus of Pin25611FI, and was also assigned as an m6A dependent R-M system targeting GGATG/CATCC through REBASE prediction and motif analysis.

The second system, Pin17FORF7650P, contains two separate orphan MTase genes (*m1* and *m2*), which were both proposed to recognize GATC through REBASE prediction. M1.Pin17FORF7650P is clearly a homolog of the MTase of the Pin25611FII R-M system in ATCC-25611F (98% coverage 95% identity between MTase proteins) and it occupies the same genetic loci, except that the REase gene has been lost in 17F. In its place, there is a 1.85 kb region of little homology, which harbors a second MTase gene, also assigned to GATC through REBASE **(Fig 4B)**. Methylation can occur at the adenine and the cytosine residues of the GATC motif and Accetto *et al* [45] previously identified an MTase which modified the cytosine residue of this motif within the related species *Prevotella bryantii* TC1–1. Accordingly, based on the presence of two separate MTase genes assigned to the GATC motif, we next investigated the possibility that both positions (GATC) are methylated in *P*. *intermedia* 17F and sought to determine if the second MTase caused m6A or m4C/m5C modifications.

We used the isoschizomeric REases Sau3AI, DpnI, DpnII and MboI to assess the methylation status of GATC sequences in the genome of *P*. *intermedia* 17F, including ATCC-25611F also in this assay for comparison, as it lacks the second MTase gene. Sau3AI cuts GATC sites regardless of their adenine methylation state but is blocked by cytosine methylation, DpnI requires adenine methylation to cut GATC but is also blocked by cytosine methylation, DpnII cuts GATC regardless of the cytosine methylation status but is blocked by adenine methylation, while MboI cuts only fully unmethylated GATC sequences. The unrelated REase ApoI, is included as a positive control, recognizes the motif RAATTY which occurs throughout the genome of *P*. *intermedia* species.

Both *P*. *intermedia* strains were entirely resistant to MboI and DpnII restriction **(Fig 5B)**, confirming that GATC sites are indeed methylated, a result of m6A modification likely caused by the activity of M. Pin25611FII and M1.Pin17FORF7650P systems *in vivo* **(Fig 5A)**. However, the resistance to digestion by DpnI suggests that the cytosine residue is also methylated, and this is also confirmed by the poor digestion with Sau3AI. Further extension of the REase assay to 12 hours incubation with an excess of each enzyme (5U/ug DNA) resulted in the same patterns of digestion for *P*. *intermedia* and we additionally established that poor digestion observed was not a result of inhibitors present during the REase reactions **(S3 Fig)**. In contrast, *F*. *nucleatum* ATCC-25586 DNA was sensitive to all enzymes except for DpnI, indicating that GATC is unmethylated in this strain. The higher levels of control ApoI digestion observed in *F*. *nucleatum* compared to *P*. *intermedia* is likely a result of lower genomic G+C content (27% versus 42% respectively) in this organism, which would contain higher levels of the A+T rich ApoI target motif.

It is interesting that both 17F and ATCC25611F gDNAs are resistant to all REase enzymes that are influenced by cytosine methylation of the GATC motif, especially considering that ATCC25611F does not have a corresponding unassigned MTase gene recognizing GATC. When M2.Pin17FORF7650P was cloned and expressed in *E*.*coli* ER2796 no protection against either Sau3AI or DpnII REase was observed **(S4 Fig)**, preventing definitive assignment of this MTase to either modification. In light of the inactivity of this second GATC gene, the *m1* gene is presumed to be active in 17F and has been named M1.Pin17FII accordingly. It remains unclear why *P*. *intermedia* gDNA appears resistant to both Sau3AI and DpnI cleavage, but bisulfite sequencing analysis confirmed that m5C modification is not present within this motif **(Fig 5C and S5 Fig)**.

A unique Type IIG R-M system, not present in ATCC-25611F, was found in 17F. Type IIG systems are composed of bi-functional enzymes with a DNA-cleavage domain and a gamma-class DNA-methylation domain in a single protein chain that cleaves at fixed distances from their recognition sequence [40, 46]. The 17F TIIG system is composed of two individual genes, *pin17FORF9590P*, and *pin17FORF9595P*, which are located on a 7.6 kbp locus nested between multiple mobile element proteins suggesting its acquisition through horizontal gene transfer **(Fig 4A)**. While each of the two proteins of this system were assigned as Type IIG REase/m6A MTases, no assignment of their specificity was possible through REBASE. However, we did note that a similar Type IIG system exists in *Rikenella microfusus* DSM-15922 which shares considerable protein homology to the two R components of the 17F system (Pin17FORF9590P—93% coverage and 65% identity and Pin17FORF9595P with 89% coverage and 62% identity, respectively). Interestingly, the methylome data available for *R*. *microfusus* also identified three unassigned motifs (CCGACY, CCRGAG, SCCAAB) which appear to be closely related variants of our 17F unassigned motifs (GGNYAG, CCNYAG and GGYGAB where Y = C or T, R = A or G and B = C or G or T). To investigate whether the Pin17FIII system was indeed responsible for these non-palindromic sequence modifications we cloned both *pin17FORF9590P* and *pin17FORF9595P* in isolation on separate constructs, as well as in a single construct **(S1 Table)**. SMRT sequencing and methylome analysis of plasmid DNA from these clones confirmed that expression of Pin17FORF9590P in *E*. *coli* ER2796 results in m6A modification of GGYGAB motifs **(S3 Table)**, and this system has been assigned as Pin17FIII in 17F. However, expression of the *pin17FORF9595P* gene in isolation did not reveal any modified motif upon SMRT sequencing, while the expression of both genes together resulted in the same m6A modification of GGYGAB but not any additional modifications. Nevertheless, the successful assignment of the *pin17FIII* gene to the GGYGAB motif and the homology of the other genes in this system to that of *R*. *microfusus* suggests that one or a combination of genes in this system, perhaps through the interaction of the upstream SNF2/RAD54 family helicase protein, may be responsible for the remaining GGNYAG and CCNYAG motifs identified during methylome analysis of 17F.

A further two orphan m6A MTases, M.Pin17FORF2975P and M.Pin17FORF8660P, were identified that are closely related to each other (90% protein coverage, 89% identity) and present on two separate, but homologous, 23 kb prophage islands (87% nucleotide coverage and 81% identity) with G+C content higher the genome average (47.9%) on chromosome II. These genes show some similarity to *m*.*wviII*, (98% nucleotide coverage and 52% identity) which recognizes the promiscuous sequence BA (B signifies not A). Due to their location within a likely prophage and the absence of another m6A modification from methylome analysis they are not expected to be expressed and we assigned these as inactive.

Lastly, the *pin17FORF11110P* gene was identified as a possible isolated Type II REase of unknown recognition sequence. This REase shares 99% coverage and 61% identity at the protein level to a *Flavobacterium columnare* REase associated with an active Type II R-M system of unknown target recognition [23] but exists in *P*. *intermedia* 17F as a solitary REase which lacks an apparent MTase partner. As such, this enzyme’s target recognition motif either overlaps with another system in 17F, or it is inactive. The gene itself occurs 5 kb downstream of a transposase and three mobile element proteins, suggesting that this isolated REase may have originally formed due to an incomplete transposition event.

Overall, in addition to a number of orphan MTase systems we identified three active R-M systems present in *P*. *intermedia* ATCC-25611F, targeting GCAGC, GATC and GGATG motifs, and two in the 17F clinical isolate, targeting GGATG and GGNYAG, CCNYAG, GGYGAB motifs, which could obstruct the uptake and incorporation of exogenous DNA in these strains during horizontal gene transfer and artificial transformation *in-vitro*.

#### 3.2.3 Type IV systems

**Fig 7:**
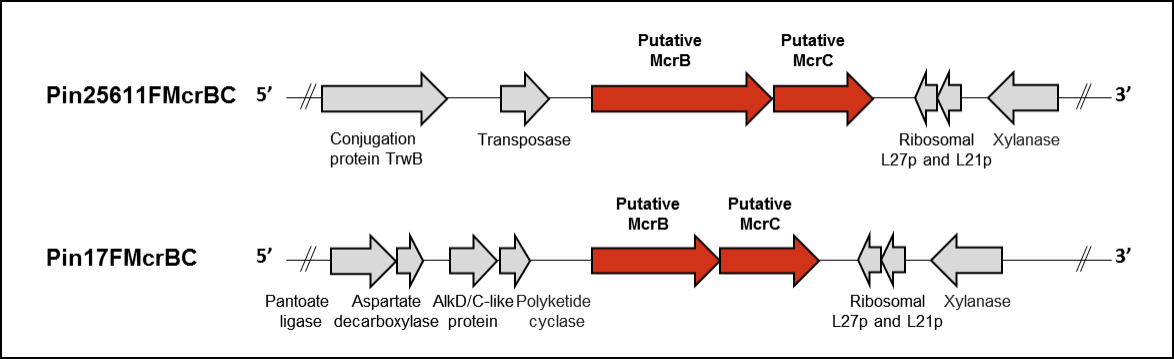
Type IV restriction systems of *P*. *intermedia* ATCC-25611F (upper panel) and 17F (lower panel). Arrows represent the direction of translation and the relative sizes of open reading frames (ORFs). Putative McrBC methyl-directed endonuclease homologs are indicated in red. ORFs not identified as part of these REase systems or those with currently unknown function are shown in grey. Predicted functions of these genes are predicted from BLAST analysis, using default settings. Exact genome coordinates of each putative McrBC loci are indicated in **Table 1**and **Table 2**.

Type IV systems are distinct from Type I and II, in that they do not utilize an MTase enzyme. Instead a methyl-dependent REase targets a non-specific and variable sequence if it contains a DNA modification, including methylated, hydroxymethylated and glucosyl-hydroxymethylated bases. These systems are exemplified by the modified cytosine restriction McrBC [47] and modified DNA rejection and restriction Mrr [48] systems of *E*. *coli*, and their presence in a bacterial host has significant implications for genetic engineering and transformation efficiency [49, 50]. We identified a putative Type IV methyl-directed REase system containing two genes in both *P*. *intermedia* ATCC-25611F and 17F **(Fig 7)**. However, the classification of Type IV systems and identification of their target recognition sequences is inherently more difficult than for Type I and II systems and cannot be determined through SMRT sequencing and methylome analysis. Currently, there are 8,210 putative Type IV REases in REBASE [23], but complete characterization of sequence preference and cleavage position has only been carried out for three, EcoKMcrBC of *E*.*coli*, SauUSI of *Staphylococcus aureus* and PvuRts1I of *Proteus vulgaris* [48]. Of these, the *P*. *intermedia* Type IV systems are most similar to the EcoKMcrBC system, which targets RmC (where R = A or G, and either m4C or m5C modification) separated by ∼40 to 3,000 bases[51]. Yet, based on the low homology of the individual protein subunits (*E*. *coli* McrB/McrC: 63% coverage with 37% identity / 41% coverage with 27% identity in ATCC-25611F or 59% coverage with 38% identity / 36% coverage with 24% identity in 17F) we are unable to accurately predict the target of these Type IV systems at this time **(Table 1**and **Table 2)**. In the context of *in vitro* transformation, unintentional activation of Type IV systems has previously been avoided by the propagation of plasmid DNA within an *E*. *coli* host that does not methylate DNA (*dam*- *dcm*- *hsdRMS*-), thus avoiding recognition and degradation via these systems [52].

### 3.3. Identification of *Prevotella intermedia* CRISPR-Cas systems and their target sequences

CRISPR defense is based on the self/non-self discrimination principle, but rather than utilizing epigenetic information like R-M systems, CRISPR-Cas imprints pieces of genetic material to the genome as a heritable memory of previously encountered invading/exogenous DNA. For a detailed review on the mechanism of CRISPR defense in bacteria, readers are referred to [53]. Briefly, CRISPR-Cas systems are composed of at least one CRISPR array and a set of *cas* (CRISPR-associated) genes. While the majority of CRISPR array spacers sequences typically match with regions of bacteriophage genomes, many match plasmids, other mobile genetic elements, and chromosomal regions of other bacteria [54–56]. Thus, CRISPR-Cas systems constitute a major barrier against the transfer of genes, accessory genetic elements, and human-made genetic tools, which are often constructed from cryptic plasmids [57], during artificial conjugation and electro-transformation of bacterial species [58–60].

Accordingly, we surveyed our *P*. *intermedia* genomes for the presence of CRISPR-Cas systems, and sought to define the specific spacers on any CRISPR array, as corresponding sequences present on human-made DNA with an adjacent PAM (protospacer adjacent motif) sequence would lead to DNA degradation by active systems. For a more extensive characterization of CRISPR-Cas systems in the context of the pan-genome and evolutionary perspective of *Prevotella intermedia* and related species, readers are directed to a recent study by Ibrahim *et al* [61].

**Fig 8:**
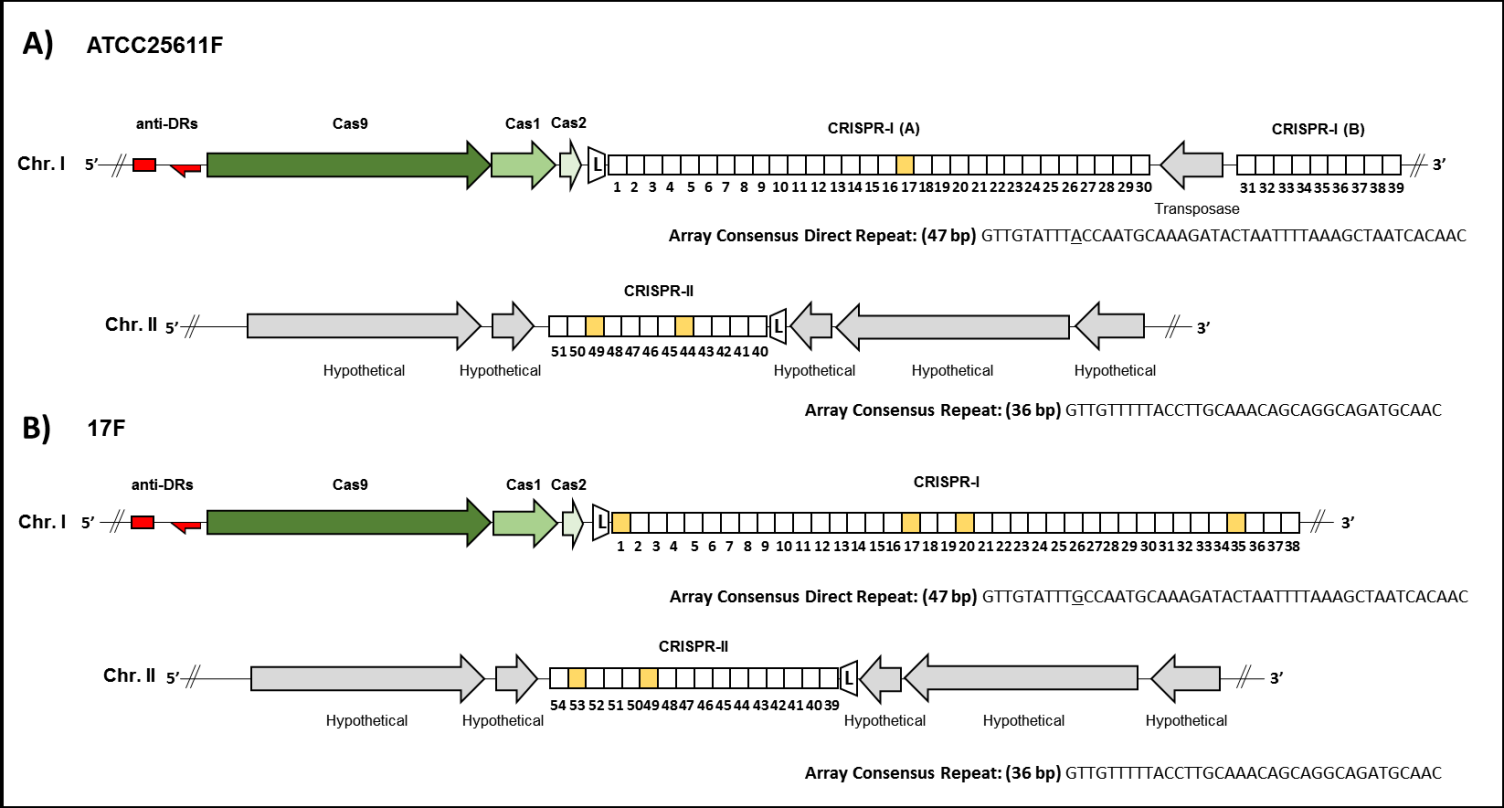
**CRISPR-Cas systems of A) *P*. *intermedia* ATCC-25611F and B) *P*. *intermedia* 17F. CRISPR loci and *cas* gene organization within *P*. *intermedia* genomes.** Arrows indicate open reading frames, with gene names indicated above/below each. Predicted *cas* genes associated with each CRISPR locus are shaded in green while ORFs not identified as part of the CRISPR system or those with currently unknown function are shown in grey. Anti-DR repeats loci are indicated in red with arrows indicating putative tracrRNA and their orientation. CRISPR arrays are represented by hatched rectangles with each square representing a single spacer sequence which we have numbered according to its position relative to the predicted leader sequence (L). Squares shaded yellow indicate that the spacer showed similarity (>87% homology) to phage or plasmids during CRISPRtarget analysis. The consensus DR sequence is indicted below each array (non-conserved nucleotides between systems are underlined). Chr. I and Chr. II indicate the location of the respective CRISPR loci on chromosome I or chromosome II of each strain, while exact genome coordinates of each component are detailed in **S4**and **S5 Tables**.

Based on their effector module organization, CRISPR-Cas systems of prokaryotes are split into two distinct classes [62, 63]. Class 1 CRISPR-Cas systems utilize multi-protein effector complexes, whereas class 2 CRISPR-Cas systems utilize single-protein effectors [62]. Analysis of ATCC-25611F and 17F genome sequences using a combination of the CRISPRdetect, CRISPROne and CRISPRfinder programs revealed two separate CRISPR systems present in both strains. The first system, located on chromosome I and designated CRISPR-I, has adjacent Cas proteins indicative of a class 2 type II-C system [64] while the second, located on chromosome II and designated CRISPR-II, is devoid of known *cas* gene homologs and is therefore assumed to be an isolated locus **(Fig 8)**.

In the *P*. *intermedia* CRISPR-I system, we noted the presence of a large (1381 amino acid) Cas9 protein coding gene homolog adjacent to the CRISPR-I loci in both strains. In class 2 type II systems, Cas9 is sufficient to both generate crRNA (CRISPR-RNA) transcript from the array and also to act as the effector nuclease and cleave target sequences during CRISPR-mediated interference of invading DNA [65]. In addition to this *cas9* gene, we also observed two adjacent genes assigned to express Cas1 and Cas2 proteins by protein BLAST, which are ubiquitous in these CRISPR types and presumed to be essential during the integration of spacers into the CRISPR array [66]. The absence of a fourth *cas* gene in this system, which would typically encode for Csn2 (in Type II-A systems) or Cas4 (in Type II-B systems) allowed us to further distinguish the *P*. *intermedia* CRISPR-I as a Type II-C system [67]. The *cas9*/*cas1*/*cas2* genes within the 5.4 kbp CRISPR-I region are highly conserved between ATCC-25611F and 17F (99% nucleotide coverage, 94% identity).

The direct repeats (DR) of the CRISPR-I array were also well conserved between strains with respect to length (47 bp) and sequence, (ATCC-25611F: GTTGTATTTACCAATGCAAAGATACTAATTTTAAAGCTAATCACAAC, and 17F: GTTGTATTTGCCAATGCAAAGATACTAATTTTAAAGCTAATCACAAC) and were interspersed with n = 39 or n = 38 individual protospacers respectively. In Type II systems, the processing of the CRISPR array transcript (pre-crRNA) into individual targeting crRNAs is dependent on the presence of a trans-activating crRNA (tracrRNA) [68]. The tracrRNAs are encoded by sequences found in close proximity to the *cas* operon and CRISPR array and are identified by the presence of an anti-repeat sequence complementary to the DR sequence [64, 69]. Hybridization of the tracrRNA to the DR sequence triggers processing by bacterial double-stranded RNA-specific ribonuclease, RNase III, and facilitates subsequent binding with Cas9 and degradation of target DNA [70]. CRISPROne analysis identified two putative tracRNA loci, anti-DR-1 and anti-DR-2 **(Fig 8)**, upstream of the *cas9* gene with homology to the DR of CRISPR-I system, present in both ATCC25611F (anti-DR-1: 80% coverage / 84% identity, anti-DR-2: 72% coverage / 92% identity) and 17F (anti-DR-1: 72% coverage / 88% identity, anti-DR-2: 73% coverage / 92% identity). One or both of these anti-repeat sequences may form the tracrRNA of the *P*. *intermedia* strains CRISPR-I system, and to investigate further we utilized the workflow developed by Chylinski *et al* [68] for *in silico* prediction of tracrRNA orthologs. Both of the putative tracRNA loci were found to occur within intergenic regions and anti-repeat:DR base-pairing of each was confirmed using multiple sequence alignment (**S6 Fig).** We therefore next screened the flanking regions (150 bp) of each anti-repeat loci for predicted promoters and Rho-independent transcriptional terminators which could be associated with tracrRNA transcription. We were unable to strongly predict promoters for either anti-DR-1 or anti-DR-2, in either orientation; however we did identify a putative Rho-independent transcriptional terminator [71] approximately 30 bp upstream of the anti-DR-2 loci, which was highly conserved in both ATCC25611F and 17F (**S6 Fig**). This would suggest that anti-DR-2 is the best candidate for the CRISPR-I tracRNA loci and that it is transcribed in the opposite direction of the *cas* operon (**Fig 8**), which has been observed previously for Type II CRISPR systems in *F*. *novicida*, *N*. *meningitidis* and *C*. *jejuni* [68].

In addition to DRs, tracRNA, and captured protospacer sequences, CRISPRs typically contain a ∼100–500 bp A-T rich leader element that typically includes a transcription promoter and indicates the orientation of the array [72]. The leader sequence lacks an ORF, has been shown to be well conserved within the same species, and also contains a palindromic sequence [73]. Alkhnbashi *et al* previously analyzed and characterized putative leader sequences for 1426 archaeal and bacterial genomes [74], including *P*. *intermedia* 17 (http://www.bioinf.uni-freiburg.de/Software/CRISPRleader/). In ATCC25611F and 17F, the orientation of the array is indicated by the 120 bp CRISPR-I leader between the *cas2* gene and the first DR of the CRISPR-I array **(Fig 8)**. The leader sequence is well conserved between strains (95% nucleotide identity) and contained an obviously long palindromic sequence, ‘TGGAACTTTATT/AATAAAGTTCCA, separated by 11 bp **(S7 Fig)**. In agreement with other characterized leader sequences [74], the CRISPR-I leader is AT rich, with a GC content of 32% compared to the genomic average of 43% for *P*. *intermedia*.

Interestingly, unlike to the relative conservation of DRs, putative tracrRNA sequences and *cas* genes between strains, we did not observe any conservation in captured protospacer sequences between ATCC-25611F and 17F **(S4**and **S5 Tables)**. Additionally, in ATCC-25611, the CRISPR-I locus has been interrupted by a transposase-encoding gene between spacer 30 and 31 **(Fig 8A)**, dividing it into CRISPR-I (A) and CRISPR-I (B), which is not found in 17F. It is notable that Naito *et al* previously reported a much larger 34.2 kb prophage-related insertion within the *cas2* gene of a homologous CRISPR system in the related clinical isolate *P*. *intermedia* OMA14, resulting in the apparent inactivation of that CRISPR system [75]. The transposon insertion within the ATCC25611F array is likely to interfere with the processing of the pre-crRNA transcript, potentially inactivating protospacers 31 to 39 in ATCC25611F, but determination of the precise impact of this transposon insertion on the function of the entire CRISPR-I system will be important to address in future studies.

The second loci CRISPR-II, occurring on chromosome II of both, is an isolated CRISPR array locus lacking known *cas* gene homologs with a highly conserved DR length (36 bp) and sequence between both strains (GTTGTTTTTACCTTGCAAACAGCAGGCAGATGCAAC) containing n = 12 and n = 16 spacers in ATCC-25611F and 17F respectively. The orientation of the array was predicted with low confidence by CRISPRdetect, based on the degeneracy in DR sequence at the 3’ end of the array **(S4 and S5 Tables)**, while no strong leader sequence was detected by CRISPRleader analysis on either flanking region. CRISPROne analysis also failed to detect any putative anti-DR/tracrRNA within the vicinity of the array however it is notable that not all CRISPR types require tracrRNA sequences to function [64]. Isolate CRISPRs have been reported in numerous bacteria including the associated oral pathogen *Aggregatibacter actinomycetemcomitans* [76]. Some have been observed to be transcribed but not processed into crRNA, which indicates they may be the remnants of previous functional CRISPR–Cas systems [77]. Although the DR of this system is shorter than the CRISPR-I locus, there is some conservation in DR sequence between CRISPR-I and -II DRs, especially in the 5’ sequence where 17 of the first 20 nt are shared, in addition to a 3’-CAAC motif **(S6 Fig)**. It remains to be demonstrated whether isolated CRISPR systems with such differences in DR sequences can function remotely with Cas proteins of other active systems within the same organism [77]. Similar to the CRISPR-I system, there does not appear to be any conservation in captured protospacer sequences between ATCC-25611F and 17F for this isolate CRISPR.

Given that protospacers play an important role in defense mechanisms against exogenous DNA uptake and incorporation, we sought to determine whether protospacers in both CRISPR-I and II contained homology to known phage or plasmid sequences. In this context, the PAM motif is a component of the invading DNA but is not included in the protospacers within the array, allowing these system to distinguish between self and non-self [78]. CRISPRTarget was used to identify putative protospacers, and hits that showed ≥87% identity (≥26/30 nt or >25/29 nt) were deemed to be significant. A total of 105 unique spacers were analyzed and of these, only 9% (9/105) were homologous to known phage, plasmid (NCBI taxid: 10239 and taxid: 36549) or prokaryote (taxid: 2157, included to screen for prophage) nucleotide sequences. No sequences found were a perfect match to protospacers, instead ranging between 87–90% homology **(S6 Table)**. We further analyzed whether the putative protospacers displayed conserved PAM sequences. The flanking sequences, 10 bp on both sides of the protospacers matches, were extracted and aligned **(S8 Fig)**. Unfortunately, whether in 5’ terminal or 3’ terminal, there was no obviously conserved motif in CRISPR-I. In CRISPR-II we did observe a conserved A motif at the -2 position of the 5’ terminal however due to the overall low number of protospacers matches (n = 5 and n = 4 respectively) and no perfect target match present **(S6 Table)**, accurate predication of either the CRISPR-I or CRISPR-II systems PAM motif is not possible at this time.

In the absence of knowledge on the specific PAM motif, homologous sequences corresponding to *P*. *intermedia* CRISPR spacers present on plasmids or linear DNA cassettes could be considered as potential targets for CRISPR mediated degradation [58] and should likely be avoided during the construction of genetic tools for engineering of either *P*. *intermedia* strain. All unique spacer sequences of both ATCC-25611F and 17F are listed in **S4**and **S5 Tables** and can be used to screen genetic tools in further studies. The creation of a genetic system for *P*. *intermedia* will also facilitate more in-depth characterization of the CRISPR-Cas systems present in these strains.

### 3.4. Genome screening for DNA degradation (DND) systems

DNA phosphorothioation systems, also known as DNA degradation (DND) systems, consist of two components: modification enzymes (DndABCDE), which label DNA by phosphothiolation (PT) and restriction cognates (DndFGH), which catalyze the formation of double-stranded breaks in non-PT-protected DNA [12]. The necessary DndABCDE proteins are encoded on a highly conserved five gene operon that is widespread across >200 bacterial and archaeal species and likely spread via horizontal gene transfer [79]. We screened the *P*. *intermedia* ATCC25611F and 17F genomes for the presence of Dnd systems using a BLASTX search against the DndABCDE proteins of 31 individual bacterial species maintained at the Database of DNA Phosphorothioation [80] (dndDB) (http://db-mml.sjtu.edu.cn/dndDB/) and found no homologs to DndB, DndC, DndD or DndE present in either strain. Additionally, we screened the protein coding genes of both genomes for the associated DndF, DndG and DndH proteins using BLASTP against the representative *Salmonella enterica* DND system proteins (accessions WP_023234366, WP_000283229 and WP_023234367 respectively) but found no significant homologs present. We conclude that neither *P*. *intermedia* ATCC25611F or 17F contain any currently known DND system.

## 4. Conclusions

In this work, we have utilized SMRT sequencing to define the complete genomes and, for the first time, the methylomes of two model *Prevotella intermedia* strains, ATCC-25611 and clinical strain 17 (here re-designated as ATCC-25611F and 17F to avoid overlap of NCBI sequence data). We did not identify DND system homologs in either genome but characterized multiple Type I, II and IV R-M systems present in both, which supports our hypothesis that this organism’s genetic intractability is maintained by robust barriers to exogenous DNA uptake. Furthermore, we found considerable heterogeneity between strains, with only two out of nine R-M systems shared between ATCC-25611F and strain 17F. Additionally, in both strains we note the presence of CRISPR systems with conserved Cas proteins but no commonality in protospacer sequences incorporated within their CRISPR arrays. While individual strain differences are not surprising considering their divergent phylogenetic relationship, in the context of developing a genetic system for *Prevotella intermedia* species, such heterogeneity in R-M and CRISPR targets underscores the necessity for our analysis on these widely used model strains in contrast to clinical isolates. Additionally, the complexity of the rearranging Type I R-M system present in ATCC25611 suggests that the clinical isolate strain 17 may be a more amenable host for genetic engineering and analysis. This work provides a crucial resource required for the development of genetic tools to overcome the genetic intractability of *P*. *intermedia*, which will permit fundamental investigations into the organism’s physiology, metabolism, and pathogenesis in human disease. Such tools will also allow further mechanistic scrutiny of this pathogens R-M and CRISPR-cas systems in future studies.

## Supporting information

S1 Fig *Prevotella intermedia* genome annotations. Subsystem distribution based on RAST annotation of individual chromosomes I and II of *Prevotella intermedia* ATCC-25611F (A and B) and Strain 17F (C and D).
(TIF)

S2 Fig Site directed bisulfite sequencing of *P*. *intermedia* ATCC-25611F demonstrates 5-methylcytosine modifications within ^5’-^GCWGC^-3’^ motifs. Multiple sequence alignment of native and bisulfite treated ATCC25611F genomic DNA. Two regions, region 1(A) and region 2 (B), were selected based on the density of GCWGC motifs present and PCR amplified using primer pairs GCWGCregion1_BS and GCWGCregion2_BS **(S1 Table)**. Identical nucleotides are marked with asterisks; similar amino acids are marked with colons. Black lines indicate the location of GCWGC motifs prior to bisulfite conversion, with no cytosine residues protected from deamination. Red lines indicate the location of GCWGC motifs prior to bisulfite conversion which were protected from deamination by the presence of 5mC modification. Clustal Omega (http://www.ebi.ac.uk/Tools/msa/clustalo/) was used to align the sequences.
(TIF)

S3 Fig Additional restriction enzyme assays to determine methylation status of 5’-GATC-3’ motifs on *P*. *intermedia* genomic DNA. **A)** Extended 12-hour incubation of *P*. *intermedia* ATCC25611F gDNA with excessive concentrations (5U/ug DNA) of restriction enzymes recognizing GATC, each with different methylation sensitivity, Lane 1, undigested control DNA; lane 2, Sau3AI (inhibited by GAT^m^C); lane 3, DpnI (methyl-directed endonuclease, requires G^m^ATC but inhibited by GAT^m^C); lane 4, DpnII (inhibited by G^m^ATC, unaffected by GAT^m^C); lane 5, MboI (inhibited by G^m^ATC and GAT^m^C) and lane 6, Sau3AI and DpnI. **B)** Confirmation that enzyme inhibitors are not preventing gDNA digestion. Each enzymatic reaction (1U enzyme/1ug DNA) was repeated in the presence of internal control pRRS plasmid DNA isolated from ER2796 (unmethylated control DNA). *P*. *intermedia* gDNA (1 ug) and pRRS plasmid (500ng) were incubated together in each +/+ reaction. Plasmid DNA was digested as expected in each case, while gDNA from both *P*. *intermedia* ATCC25611F and 17F remained undigested. BfuCI was also included as it is an isoschizomer of Sau3AI, (inhibited by GAT^m^C only).
(TIF)

S4 Fig Restriction enzyme assay to determine methylation status of ^5’-^GATC^-3’^ motifs on plasmid DNA isolated from *E*.*coli* ER2796: M2.Pin17FORF7650P+. The methyltransferase gene was cloned to plasmid pRRS (primers listed in **S1 Table**) and expressed in *E*. *coli* ER2796, a strain deficient in methyltransferase activity. Plasmid DNA (1 μg) isolated from recombinant *E*. *coli* and restricted with 1U of either Sau3AI (inhibited by GAT^m^C, unaffected by G^m^ATC) or DpnII (inhibited by G^m^ATC, unaffected by GAT^m^C). In gel image: lane 1, undigested control plasmid DNA; lane 2, Sau3AI digested plasmid DNA; lane 4, DpnII digested plasmid DNA; and lane M, marker DNA (10kb ladder). No protection from REase enzyme digestion was observed.
(TIF)

S5 Fig Site directed bisulfite sequencing of *P*. *intermedia* ATCC-25611F indicates the absence of 5-methylcytosine modifications within ^5’-^GATC^-3’^ motifs. Multiple sequence alignment of native and bisulfite treated ATCC25611F genomic DNA. Two regions, region 1(A) and region 2 (B), were selected based on the density of GATC motifs present and PCR amplified using primer pairs GATCregion1_BS and GATCregion2_BS **(S1 Table)**. Identical nucleotides are marked with asterisks; similar amino acids are marked with colons. Black lines indicate the location of GATC motifs prior to bisulfite conversion, with no cytosine residues protected from deamination. Clustal Omega (http://www.ebi.ac.uk/Tools/msa/clustalo/) was used to align the sequences.
(TIF)

S6 Fig Multiple sequence alignment of CRISPR direct repeats (DR) and anti-repeats in *P*. *intermedia* strains. **A)** Comparison of ATCC25611F and 17F CRISPR-I system DR sequences. **B)** Sequence alignment of CRISPR-I system DR with putative tracRNA sites anti-repeat_1 and anti-repeat_2, identified by CRISPROne (http://omics.informatics.indiana.edu/CRISPRone). **C)** Sequence alignment of putative Rho-independent terminator of CRISPR-I tracrRNA (anti-repeat 2) in *P*. *intermedia* strains. The putative RNA secondary structure (Quikfold: http://unafold.rna.albany.edu/?q=DINAMelt/Quickfold) of the terminator sequence containing a G-C rich stem-loop is indicated in red. **D)** Sequence alignment of CRISPR-I and CRISPR-II system DRs in ATCC25611F. Identical nucleotides are marked with asterisks. Clustal Omega was used to align the sequences.
(TIF)

S7 Fig Analysis of *P*. *intermedia* CRISPR-I leader sequences. **A)** Multiple sequence alignment of putative leader sequences and the first repeats in *P*. *intermedia*. The putative leader sequence of *P*. *intermedia* (120 bp) and the first repeat (47 bp) of the CRISPR-I locus is shown. The conserved palindromic sequence is highlight in red, the first DR of the array is highlighted in blue. Identical nucleotides are marked with asterisks; similar amino acids are marked with colons. **B)** The putative secondary structure of the CRISPR-I leader sequences containing the characteristic palindromic stem-loop structure up stream of the first direct repeat. Clustal Omega was used to align the sequences and secondary structure prediction of the leader was performed using Mfold (http://unafold.rna.albany.edu/?q=mfold/RNA-Folding-Form).
(TIF)

S8 Fig Prediction of PAM consensus sequence. Putative protospacers flanking sequence (10 bp on each side, 5’: -1 to-10 and 3’:+1 to +10) were extracted and aligned. The alignment of these regions was used to create the sequence logo by WebLogo (http://weblogo.berkeley.edu/logo.cgi) for CRISPR-I and CRISPR-II of *P*. *intermedia* strains. The height of the letters indicates the relative frequency of the corresponding base at that position **A)** In CRISPR-I, no obviously conserved motif was observed in the 5’ or 3’ terminal. **B)** In CRISPR-II, alignment of flanking regions of n = 4 putative protospacers with 87% identity to spacers in this array reveled a potentially conserved adenine (red line) at -2 position of the 5’ terminal. PAM: protospacer adjacent motif.
(TIF)

S1 Table Oligonucleotides used in this study. Primers are shown in 5’ to 3’ orientation.
(XLSX)

S2 Table Methylated motifs identified in *Prevotella intermedia* ATCC-25611F and 17F. *a* The modified base within each motif is bolded while the modified base in the complementary strand is italicized. *b* Novel recognition sequences. *c* Detected only after tet-assisted SMRT library preparation. *d* The total number includes motifs occurring on the “+” and “–” strands. *e* Low percentage detected, due to m5C modification.
(XLSX)

S3 Table PacBio SMRT sequence basemod motif summary of recombinant *E*. *coli* ER2796 clones expressing *P*. *intermedia* R-M system methyltranserase genes. *motifString*: Detected motif sequence. *centerPos*: Position in motif of modification. *fraction*: Fraction of instances of this motif with modification Quality Value (QV) above the QV threshold. *nDetected*: Number of instances of this motif with above threshold. *nGenome*: Number of instances of this motif in reference sequence. *groupTag*: A string identifying the motif grouping. For paired motifs this is “/”, For unpaired motifs this equals motifString. *partnerMotifString*: motifString of paired motif (motif with reverse-complementary motifString). *meanScore*: Mean Modification QV of detected instances. *meanIpdRatio*: Mean interpulse duration (IPD) ratio of detected instances. *meanCoverage*: Mean coverage of detected instances. *objectiveScore*: Objective score of this motif in the motif finder algorithm.
(XLSX)

S4 Table CRISPR systems spacers of *P*. *intermedia* ATCC-25611F. (XLSX)

S5 Table CRISPR systems spacers of *P*. *intermedia* 17F. (XLSX)

S6 Table CRISPR systems protospacers of *P*. *intermedia* ATCC25611F and 17F. (XLSX)
