# Supplementary figures and images for: Restriction-modification mediated barriers to exogenous DNA uptake and incorporation employed by Prevotella intermedia
Source: PLoS One. 2017 Sep 21;12(9):e0185234. doi: 10.1371/journal.pone.0185234 (PMC5608340; doi:10.1371/journal.pone.0185234)

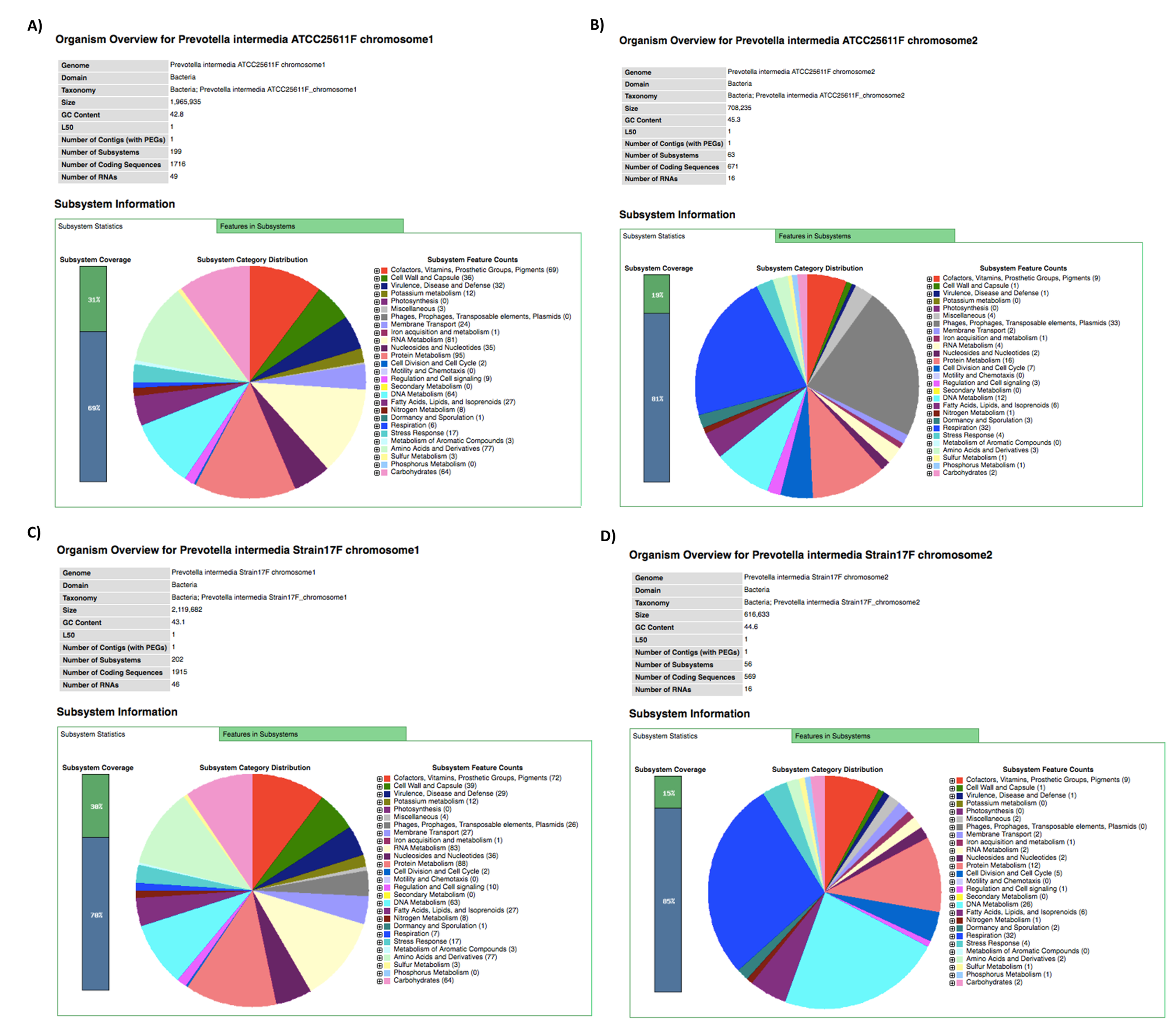

Supplement: S1 Fig — Subsystem distribution based on RAST annotation of individual chromosomes I and II of Prevotella intermedia ATCC-25611F (A and B) and Strain 17F (C and D). (TIF) [file pone.0185234.s001.tif]

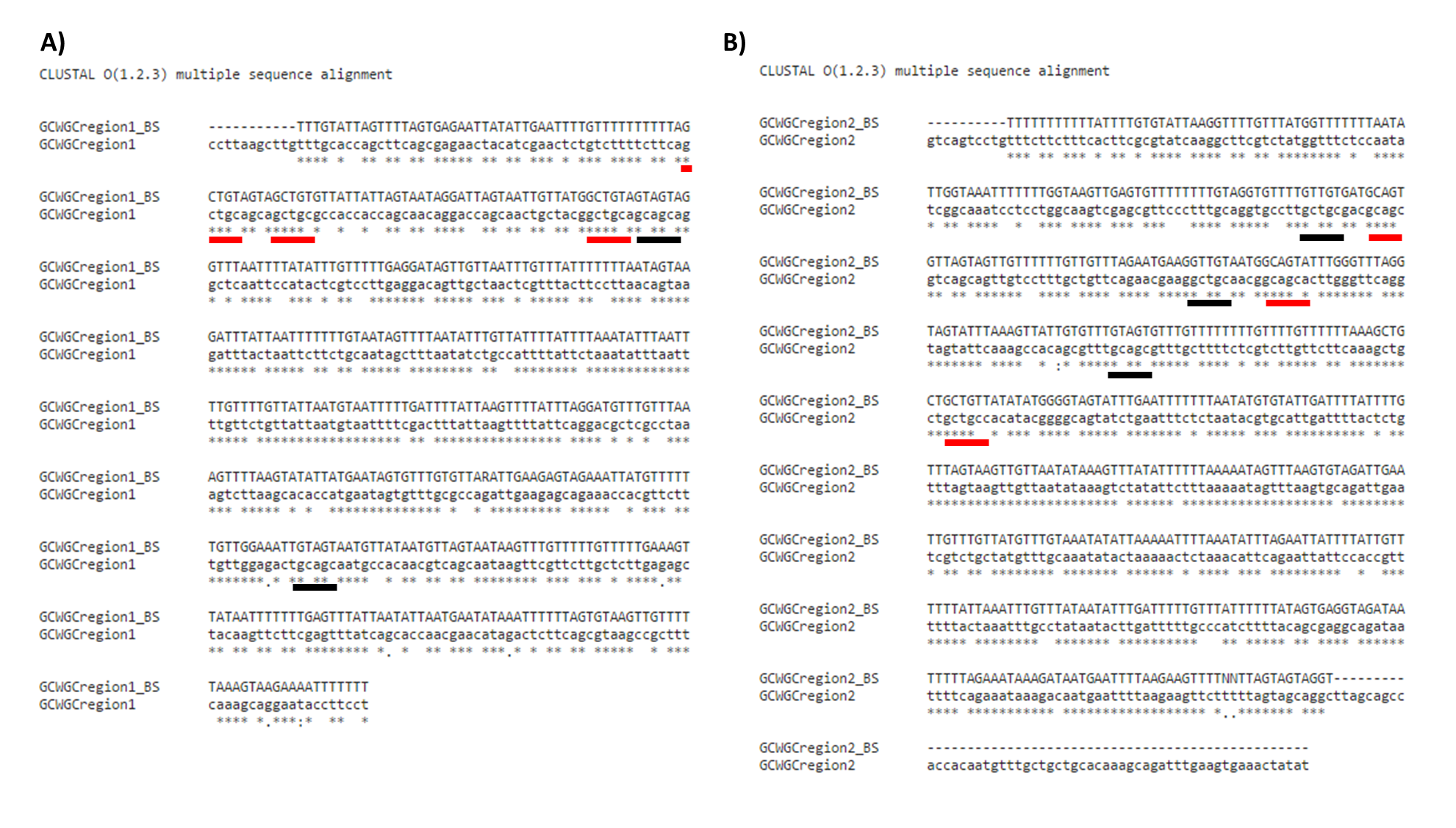

Supplement: S2 Fig — Multiple sequence alignment of native and bisulfite treated ATCC25611F genomic DNA. Two regions, region 1(A) and region 2 (B), were selected based on the density of GCWGC motifs present and PCR amplified using primer pairs GCWGCregion1_BS and GCWGCregion2_BS (S1 Table). Identical nucleotides are marked with asterisks; similar amino acids are marked with colons. Black lines indicate the location of GCWGC motifs prior to bisulfite conversion, with no cytosine residues protected from deamination. Red lines indicate the location of GCWGC motifs prior to bisulfite conversion which were protected from deamination by the presence of 5mC modification. Clustal Omega (http://www.ebi.ac.uk/Tools/msa/clustalo/) was used to align the sequences. (TIF) [file pone.0185234.s002.tif]

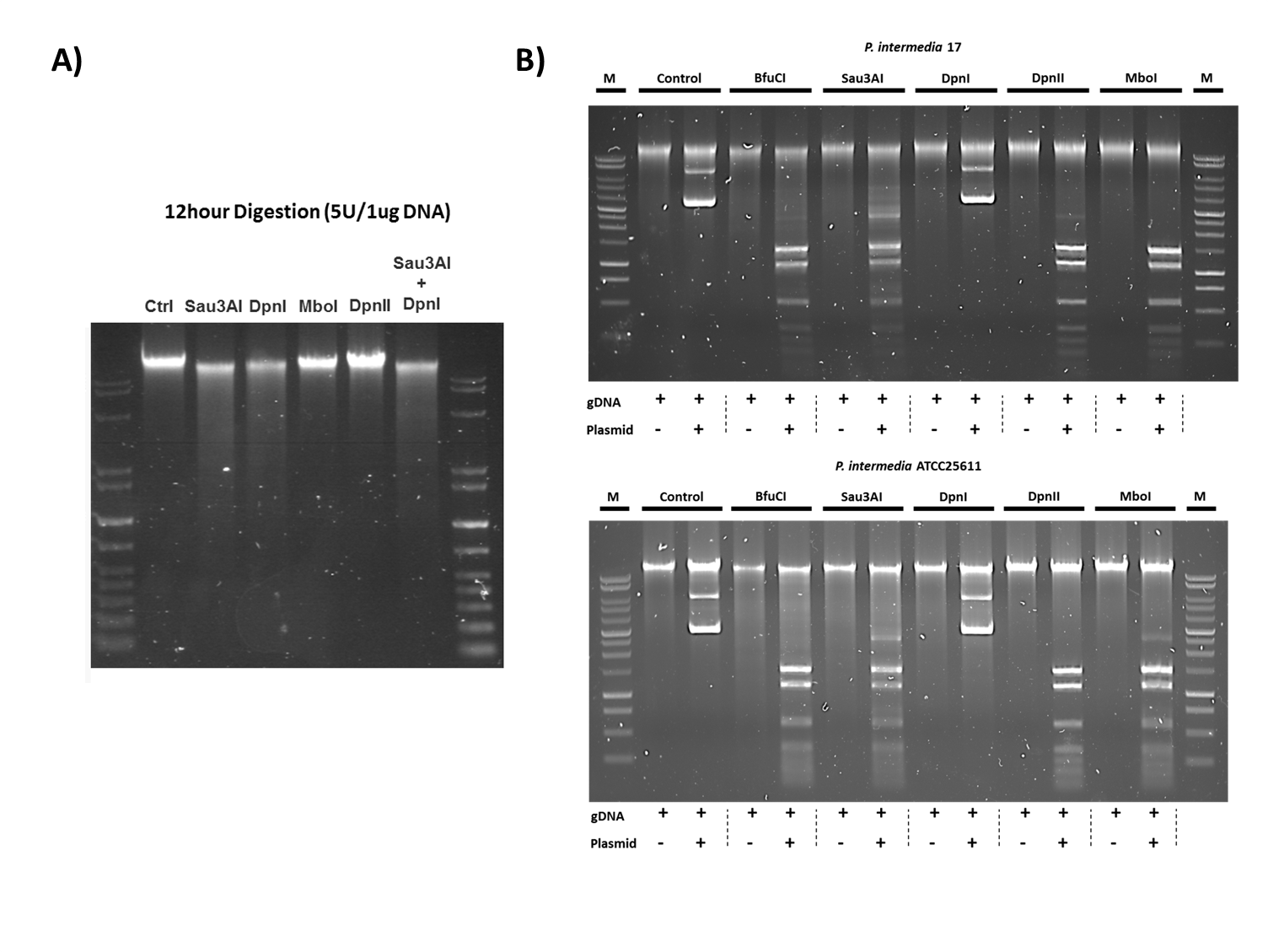

Supplement: S3 Fig — A) Extended 12-hour incubation of P. intermedia ATCC25611F gDNA with excessive concentrations (5U/ug DNA) of restriction enzymes recognizing GATC, each with different methylation sensitivity, Lane 1, undigested control DNA; lane 2, Sau3AI (inhibited by GATmC); lane 3, DpnI (methyl-directed endonuclease, requires GmATC but inhibited by GATmC); lane 4, DpnII (inhibited by GmATC, unaffected by GATmC); lane 5, MboI (inhibited by GmATC and GATmC) and lane 6, Sau3AI and DpnI. B) Confirmation that enzyme inhibitors are not preventing gDNA digestion. Each enzymatic reaction (1U enzyme/1ug DNA) was repeated in the presence of internal control pRRS plasmid DNA isolated from ER2796 (unmethylated control DNA). P. intermedia gDNA (1 ug) and pRRS plasmid (500ng) were incubated together in each +/+ reaction. Plasmid DNA was digested as expected in each case, while gDNA from both P. intermedia ATCC25611F and 17F remained undigested. BfuCI was also included as it is an isoschizomer of Sau3AI, (inhibited by GATmC only). (TIF) [file pone.0185234.s003.tif]

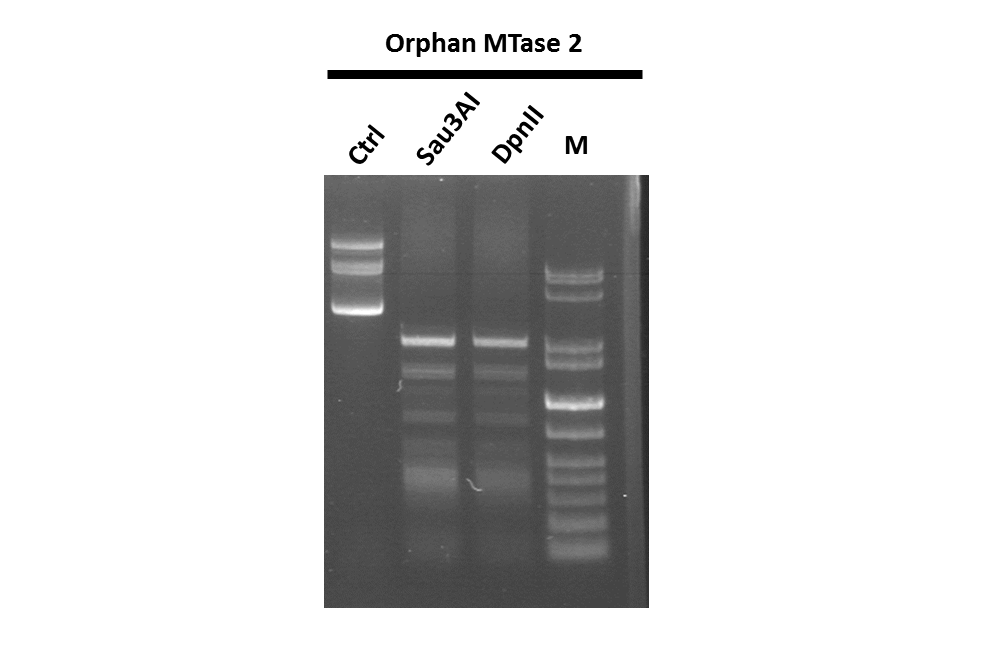

Supplement: S4 Fig — The methyltransferase gene was cloned to plasmid pRRS (primers listed in S1 Table) and expressed in E. coli ER2796, a strain deficient in methyltransferase activity. Plasmid DNA (1 μg) isolated from recombinant E. coli and restricted with 1U of either Sau3AI (inhibited by GATmC, unaffected by GmATC) or DpnII (inhibited by GmATC, unaffected by GATmC). In gel image: lane 1, undigested control plasmid DNA; lane 2, Sau3AI digested plasmid DNA; lane 4, DpnII digested plasmid DNA; and lane M, marker DNA (10kb ladder). No protection from REase enzyme digestion was observed. (TIF) [file pone.0185234.s004.tif]

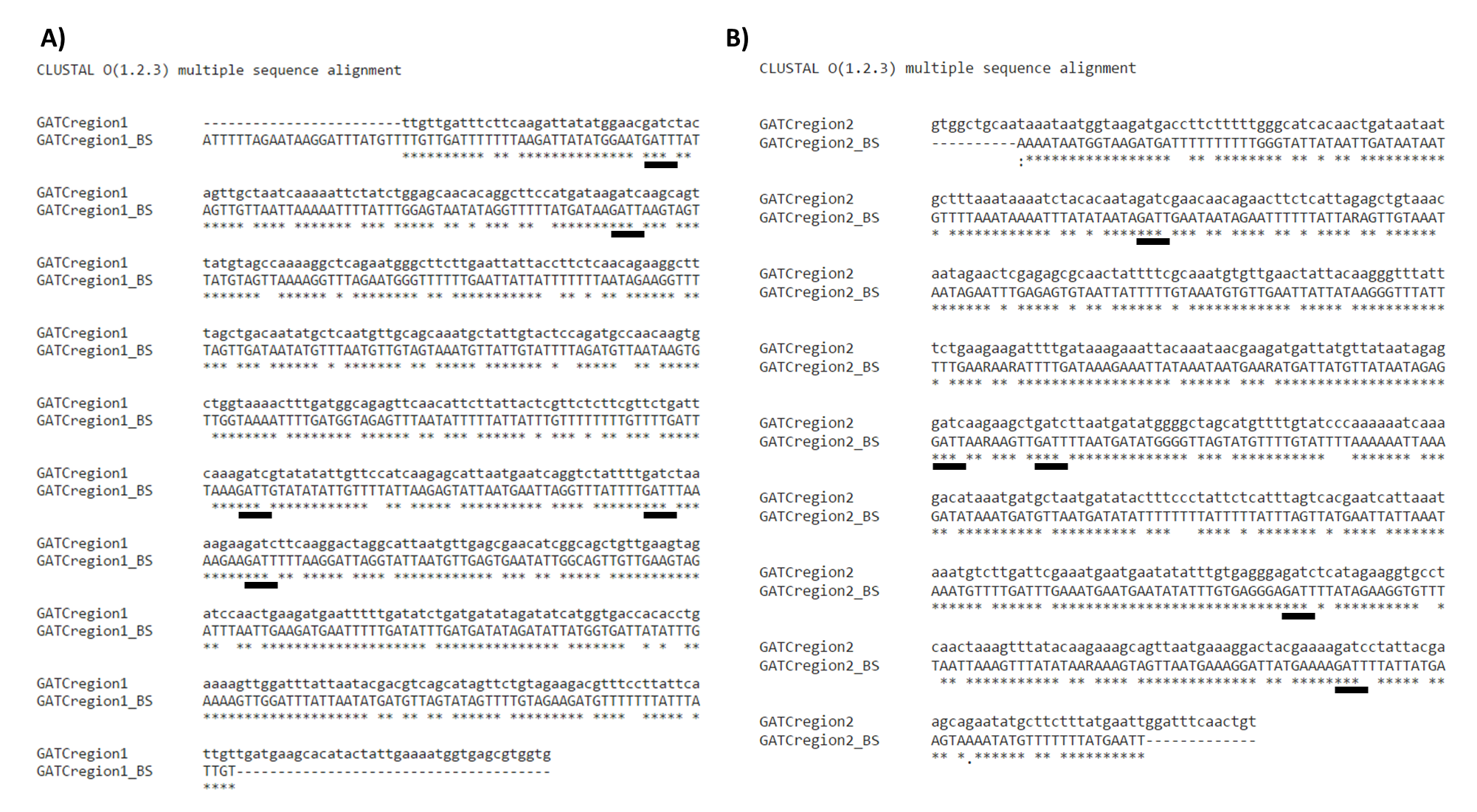

Supplement: S5 Fig — Multiple sequence alignment of native and bisulfite treated ATCC25611F genomic DNA. Two regions, region 1(A) and region 2 (B), were selected based on the density of GATC motifs present and PCR amplified using primer pairs GATCregion1_BS and GATCregion2_BS (S1 Table). Identical nucleotides are marked with asterisks; similar amino acids are marked with colons. Black lines indicate the location of GATC motifs prior to bisulfite conversion, with no cytosine residues protected from deamination. Clustal Omega (http://www.ebi.ac.uk/Tools/msa/clustalo/) was used to align the sequences. (TIF) [file pone.0185234.s005.tif]

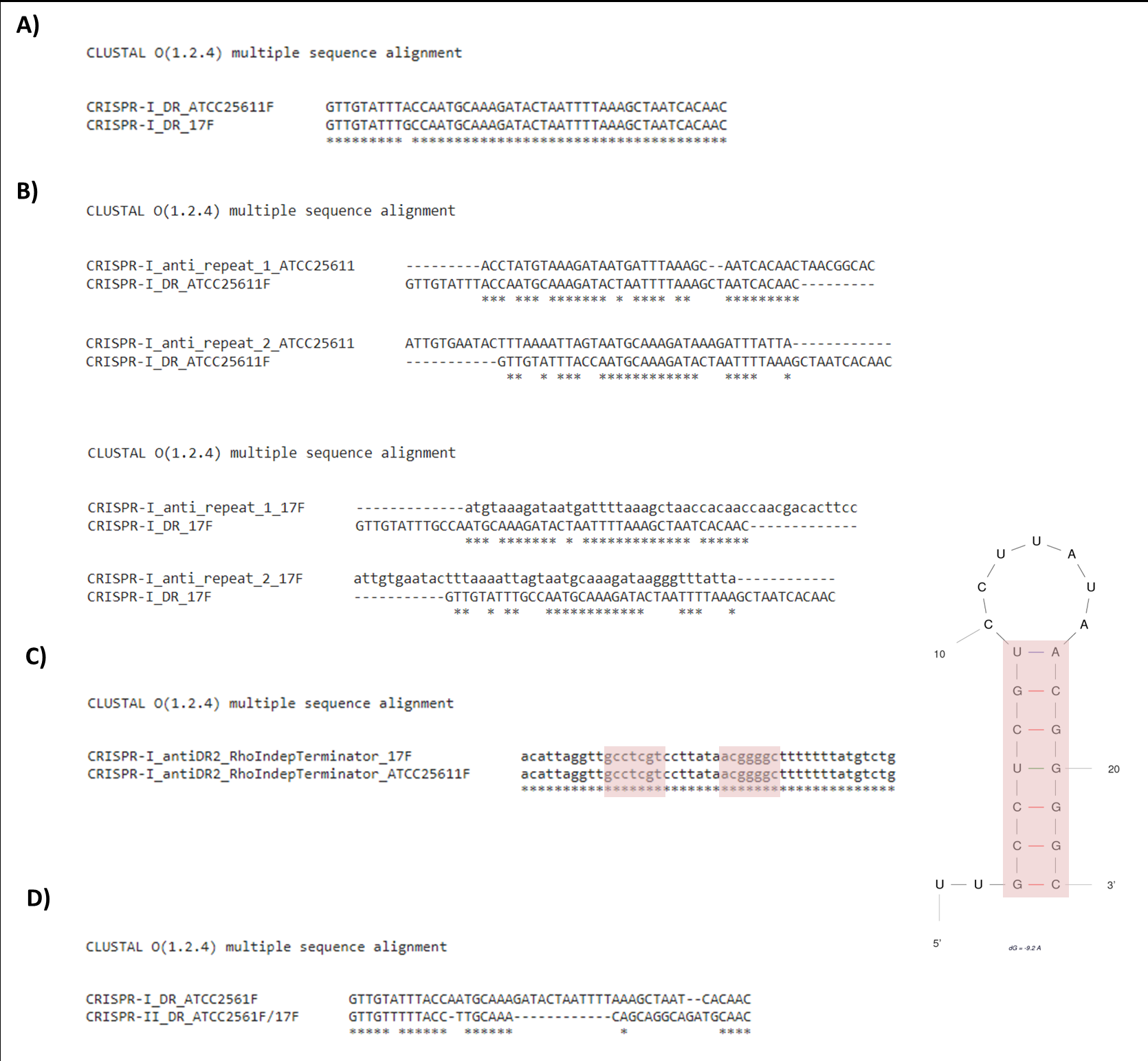

Supplement: S6 Fig — A) Comparison of ATCC25611F and 17F CRISPR-I system DR sequences. B) Sequence alignment of CRISPR-I system DR with putative tracRNA sites anti-repeat_1 and anti-repeat_2, identified by CRISPROne (http://omics.informatics.indiana.edu/CRISPRone). C) Sequence alignment of putative Rho-independent terminator of CRISPR-I tracrRNA (anti-repeat 2) in P. intermedia strains. The putative RNA secondary structure (Quikfold: http://unafold.rna.albany.edu/?q=DINAMelt/Quickfold) of the terminator sequence containing a G-C rich stem-loop is indicated in red. D) Sequence alignment of CRISPR-I and CRISPR-II system DRs in ATCC25611F. Identical nucleotides are marked with asterisks. Clustal Omega was used to align the sequences. (TIF) [file pone.0185234.s006.tif]

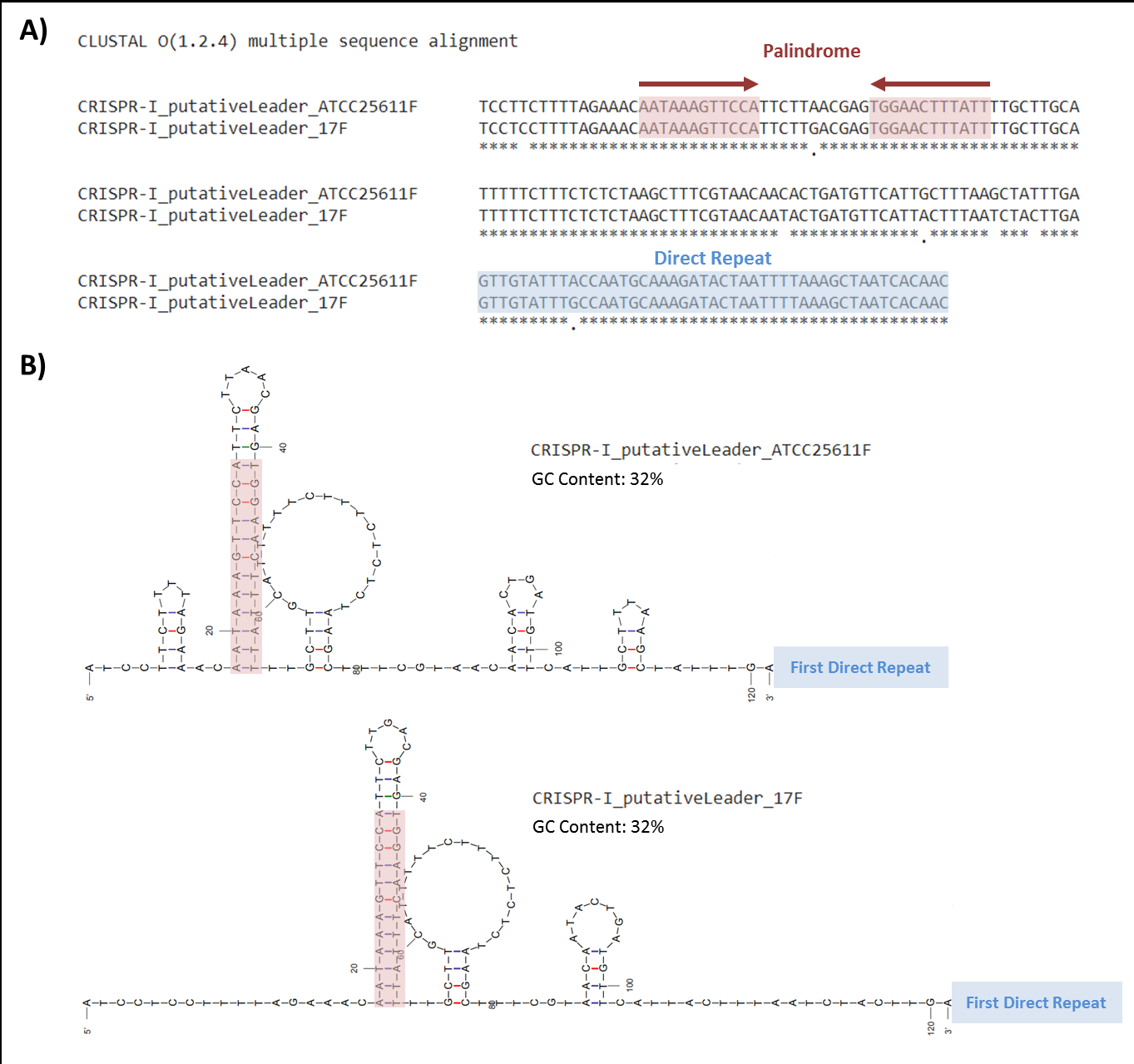

Supplement: S7 Fig — A) Multiple sequence alignment of putative leader sequences and the first repeats in P. intermedia. The putative leader sequence of P. intermedia (120 bp) and the first repeat (47 bp) of the CRISPR-I locus is shown. The conserved palindromic sequence is highlight in red, the first DR of the array is highlighted in blue. Identical nucleotides are marked with asterisks; similar amino acids are marked with colons. B) The putative secondary structure of the CRISPR-I leader sequences containing the characteristic palindromic stem-loop structure up stream of the first direct repeat. Clustal Omega was used to align the sequences and secondary structure prediction of the leader was performed using Mfold (http://unafold.rna.albany.edu/?q=mfold/RNA-Folding-Form). (TIF) [file pone.0185234.s007.tif]

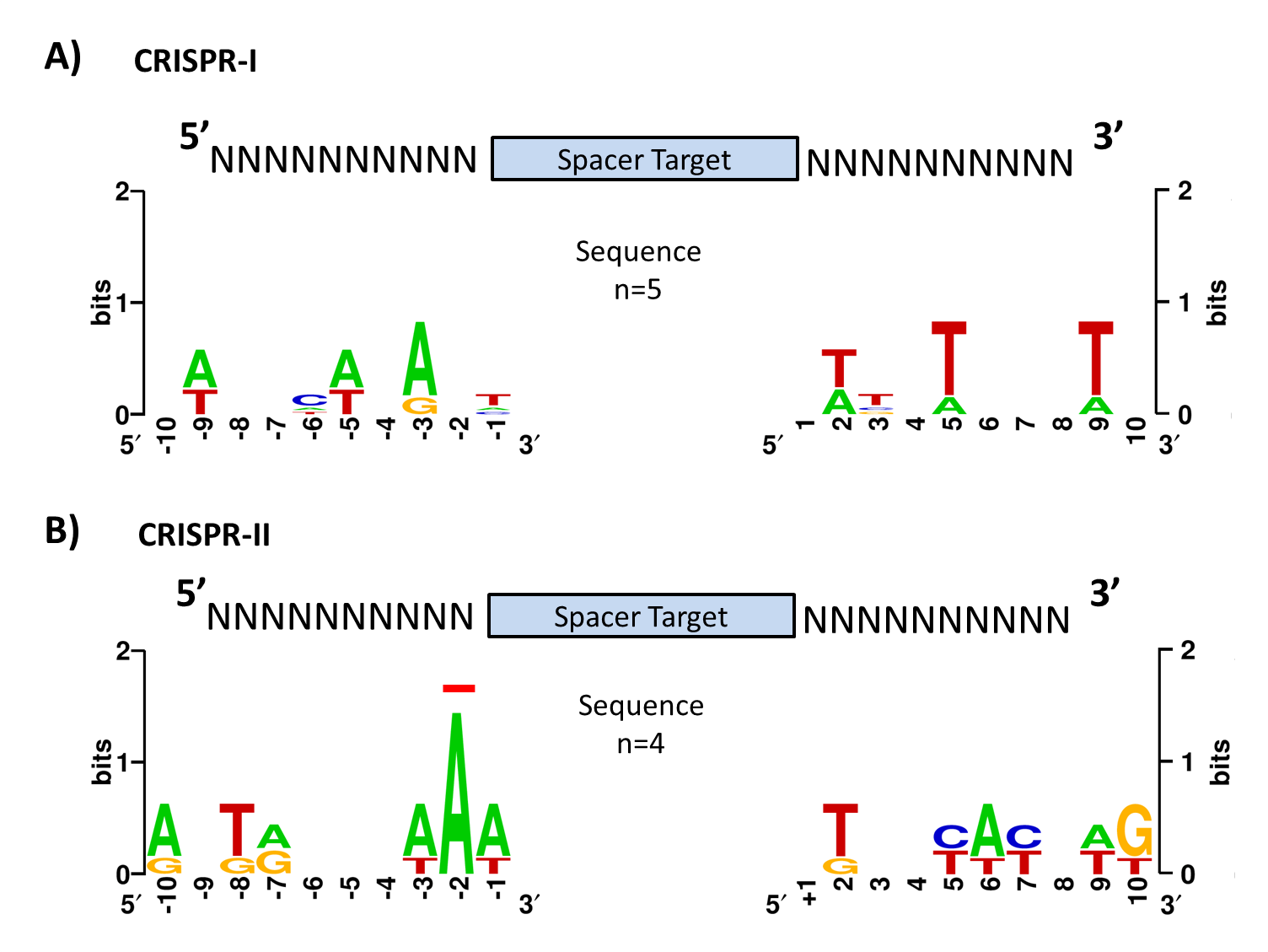

Supplement: S8 Fig — Putative protospacers flanking sequence (10 bp on each side, 5’: -1 to-10 and 3’:+1 to +10) were extracted and aligned. The alignment of these regions was used to create the sequence logo by WebLogo (http://weblogo.berkeley.edu/logo.cgi) for CRISPR-I and CRISPR-II of P. intermedia strains. The height of the letters indicates the relative frequency of the corresponding base at that position A) In CRISPR-I, no obviously conserved motif was observed in the 5’ or 3’ terminal. B) In CRISPR-II, alignment of flanking regions of n = 4 putative protospacers with 87% identity to spacers in this array reveled a potentially conserved adenine (red line) at -2 position of the 5’ terminal. PAM: protospacer adjacent motif. (TIF) [file pone.0185234.s008.tif]
